# *De Novo* and Inherited Loss-of-Function Variants in *TLK2*: Clinical and Genotype-Phenotype Evaluation of a Distinct Neurodevelopmental Disorder

**DOI:** 10.1016/j.ajhg.2018.04.014

**Published:** 2018-05-31

**Authors:** Margot R.F. Reijnders, Kerry A. Miller, Mohsan Alvi, Jacqueline A.C. Goos, Melissa M. Lees, Anna de Burca, Alex Henderson, Alison Kraus, Barbara Mikat, Bert B.A. de Vries, Bertrand Isidor, Bronwyn Kerr, Carlo Marcelis, Caroline Schluth-Bolard, Charu Deshpande, Claudia A.L. Ruivenkamp, Dagmar Wieczorek, The Deciphering Developmental Disorders Study, Diana Baralle, Edward M. Blair, Hartmut Engels, Hermann-Josef Lüdecke, Jacqueline Eason, Gijs W.E. Santen, Jill Clayton-Smith, Kate Chandler, Katrina Tatton-Brown, Katelyn Payne, Katherine Helbig, Kelly Radtke, Kimberly M. Nugent, Kirsten Cremer, Tim M. Strom, Lynne M. Bird, Margje Sinnema, Maria Bitner-Glindzicz, Marieke F. van Dooren, Marielle Alders, Marije Koopmans, Lauren Brick, Mariya Kozenko, Megan L. Harline, Merel Klaassens, Michelle Steinraths, Nicola S. Cooper, Patrick Edery, Patrick Yap, Paulien A. Terhal, Peter J. van der Spek, Phillis Lakeman, Rachel L. Taylor, Rebecca O. Littlejohn, Rolph Pfundt, Saadet Mercimek-Andrews, Alexander P.A. Stegmann, Sarina G. Kant, Scott McLean, Shelagh Joss, Sigrid M.A. Swagemakers, Sofia Douzgou, Steven A. Wall, Sébastien Küry, Eduardo Calpena, Nils Koelling, Simon J. McGowan, Stephen R.F. Twigg, Irene M.J. Mathijssen, Christoffer Nellaker, Han G. Brunner, Andrew O.M. Wilkie

**Affiliations:** 1 Department of Human Genetics, Donders Institute for Brain, Cognition, and Behaviour, Radboud University Medical Center, Nijmegen, 6500 HB, the Netherlands; 2 Clinical Genetics Group, MRC Weatherall Institute of Molecular Medicine, University of Oxford, John Radcliffe Hospital, Oxford OX3 9DS, UK; 3 Visual Geometry Group, Department of Engineering Science, University of Oxford, Oxford OX1 2JD, UK; 4 Department of Plastic and Reconstructive Surgery, Erasmus MC, University Medical Center Rotterdam, PO Box 2040, 3000 CA Rotterdam, the Netherlands; 5 Department of Clinical Genetics, Great Ormond Street Hospital, London WC1N 3JH, UK; 6 Oxford Centre for Genomic Medicine, Oxford University Hospitals NHS Foundation Trust, Oxford OX3 7HE, UK; 7 Northern Genetics Service, Newcastle upon Tyne Hospitals NHS Foundation Trust, Newcastle upon Tyne NE1 3BZ, UK; 8 Yorkshire Regional Genetics Service, Chapel Allerton Hospital, Leeds LS7 4SA, UK; 9 Institut für Humangenetik, Universitätsklinikum Essen, Universität Duisburg-Essen, 45147 Essen, Germany; 10 CHU de Nantes, Service de Génétique Médicale, Nantes 44093 Cedex 1, France; 11 INSERM, UMR-S 957, 1 Rue Gaston Veil, Nantes 44035, France; 12 Division of Evolution and Genomic Sciences, School of Biological Sciences, University of Manchester, Manchester M13 9PL, UK; 13 Manchester Centre for Genomic Medicine, Manchester University Hospitals NHS Foundation Trust, Manchester Academic Health Sciences Centre, Manchester M13 9WL, UK; 14 Department of Human Genetics, Radboud University Medical Center, Nijmegen 6500 HB, the Netherlands; 15 Hospices Civils de Lyon, Service de Génétique, Centre de Référence Anomalies du Développement, 69500 Bron, France; 16 INSERM U1028, CNRS UMR5292, UCB Lyon 1, Centre de Recherche en Neurosciences de Lyon, GENDEV Team, 69500 Bron, France; 17 South East Thames Regional Genetics Service, Guy’s Hospital, London SE1 9RT, UK; 18 Department of Clinical Genetics, Leiden University Medical Center, 2300 RC Leiden, the Netherlands; 19 Institute of Human Genetics, Heinrich-Heine-University, Medical Faculty, 40225 Düsseldorf, Germany; 20 Wellcome Trust Sanger Institute, Hinxton CB10 1SA, UK; 21 Human Development and Health, Duthie Building, University of Southampton, Southampton SO16 6YD, UK; 22 Wessex Clinical Genetics Service, Princess Anne Hospital, Southampton SO16 5YA, UK; 23 Institute of Human Genetics, University of Bonn, School of Medicine & University Hospital Bonn, 53127 Bonn, Germany; 24 Nottingham Regional Genetics Service, City Hospital Campus, Nottingham University Hospitals NHS Trust, Hucknall Road, Nottingham NG5 1PB, UK; 25 Southwest Thames Regional Genetics Centre, St George’s University Hospitals NHS Foundation Trust, St George’s University of London, London SW17 0RE, UK; 26 Riley Hospital for Children, Indianapolis, Indiana, IN 46202, USA; 27 Division of Clinical Genomics, Ambry Genetics, Aliso Viejo, CA 92656, USA; 28 Department of Pediatrics, Baylor College of Medicine, The Children’s Hospital of San Antonio, San Antonio, TX 78207, USA; 29 Department of Molecular and Human Genetics, Baylor College of Medicine, Houston, TX 77030, USA; 30 Institute of Human Genetics, Helmholtz Zentrum München, 85764 Neuherberg, Germany; 31 Institute of Human Genetics, Technische Universität München, 81675 Munich, Germany; 32 University of California, San Diego, Department of Pediatrics; Genetics and Dysmorphology, Rady Children’s Hospital San Diego, San Diego, CA 92123, USA; 33 Department of Clinical Genetics and School for Oncology & Developmental Biology (GROW), Maastricht University Medical Center, Maastricht 6229 ER, the Netherlands; 34 Genetics and Genomic Medicine, UCL Great Ormond Street Institute of Child Health, 30 Guilford Street, London WC1N 1EH, UK; 35 Department of Clinical Genetics, Erasmus MC, University Medical Center Rotterdam, PO Box 21455, 3001 AL Rotterdam, the Netherlands; 36 Department of Clinical Genetics, Academic Medical Center, PO Box 22660, 1100 DD Amsterdam, the Netherlands; 37 Department of Genetics, University Medical Center Utrecht, 3508 AB Utrecht, the Netherlands; 38 Division of Genetics, Department of Pediatrics, McMaster Children’s Hospital, McMaster University, Hamilton, ON L8N 3Z5, Canada; 39 Department of Paediatrics, Maastricht University Medical Center, Maastricht 6229 ER, the Netherlands; 40 Department of Medical Genetics, University of British Columbia, Vancouver, BC V8Z 6R5, Canada; 41 West Midlands Regional Clinical Genetics Unit, Birmingham Women’s & Children’s NHS Foundation Trust, Mindelsohn Way, Birmingham B15 2TG, UK; 42 Genetic Health Service New Zealand, Auckland 1142, New Zealand; 43 Victorian Clinical Genetic Services, Murdoch Children’s Research Institute, Melbourne, VIC 3052, Australia; 44 University of Auckland, Auckland 1142, New Zealand; 45 Department of Pathology & Department of Bioinformatics, Erasmus MC, University Medical Center Rotterdam, PO Box 2040, 3000 CA Rotterdam, the Netherlands; 46 Division of Clinical and Metabolic Genetics, Department of Pediatrics, University of Toronto, Toronto, ON, Canada; Genetics and Genome Biology Program, Research Institute, The Hospital for Sick Children, Toronto, ON, Canada; Institute of Medical Sciences, University of Toronto, Toronto, ON M5G 1X8, Canada; 47 West of Scotland Clinical Genetics Service, Queen Elizabeth University Hospital, Glasgow G51 4TF, UK; 48 Craniofacial Unit, Oxford University Hospitals NHS Trust, John Radcliffe Hospital, Oxford OX3 9DU, UK; 49 CHU de Nantes, Service de Génétique Médicale, 44093 Nantes Cedex 1, France; 50 Computational Biology Research Group, MRC Weatherall Institute of Molecular Medicine, University of Oxford, John Radcliffe Hospital, Oxford OX3 9DS, UK; 51 Nuffield Department of Women’s and Reproductive Health, University of Oxford, Women’s Centre, John Radcliffe Hospital, Oxford OX3 9DS, UK; 52 Institute of Biomedical Engineering, Department of Engineering Science, University of Oxford, Oxford OX3 7FZ, UK; 53 Big Data Institute, Li Ka Shing Centre for Health Information and Discovery, University of Oxford, Oxford OX3 7FZ, UK

**Keywords:** Tousled-like, kinase, haploinsufficiency, facial averaging, intellectual disability

## Abstract

Next-generation sequencing is a powerful tool for the discovery of genes related to neurodevelopmental disorders (NDDs). Here, we report the identification of a distinct syndrome due to *de novo* or inherited heterozygous mutations in Tousled-like kinase 2 (*TLK2*) in 38 unrelated individuals and two affected mothers, using whole-exome and whole-genome sequencing technologies, matchmaker databases, and international collaborations. Affected individuals had a consistent phenotype, characterized by mild-borderline neurodevelopmental delay (86%), behavioral disorders (68%), severe gastro-intestinal problems (63%), and facial dysmorphism including blepharophimosis (82%), telecanthus (74%), prominent nasal bridge (68%), broad nasal tip (66%), thin vermilion of the upper lip (62%), and upslanting palpebral fissures (55%). Analysis of cell lines from three affected individuals showed that mutations act through a loss-of-function mechanism in at least two case subjects. Genotype-phenotype analysis and comparison of computationally modeled faces showed that phenotypes of these and other individuals with loss-of-function variants significantly overlapped with phenotypes of individuals with other variant types (missense and C-terminal truncating). This suggests that haploinsufficiency of TLK2 is the most likely underlying disease mechanism, leading to a consistent neurodevelopmental phenotype. This work illustrates the power of international data sharing, by the identification of 40 individuals from 26 different centers in 7 different countries, allowing the identification, clinical delineation, and genotype-phenotype evaluation of a distinct NDD caused by mutations in *TLK2.*

## Main Text

The introduction of whole-exome sequencing (WES) as a diagnostic test for individuals with unexplained neurodevelopmental disorders (NDDs) has led to the identification of dozens of disease-associated genes. As a recent example, statistical analysis of aggregated exome data uncovered variants in ten different genes as likely causes of intellectual disability, a subtype of NDDs characterized by deficits in both intellectual and adaptive functioning.^1^, ^2^ One such gene was Tousled-like kinase 2 (*TLK2* [MIM: 608439]), which was originally named because of homology to the *Arabidopsis* gene *Tousled*.^3^ *TLK2*, ubiquitously expressed in all tissues including fetal brain, encodes a serine/threonine kinase comprising a catalytic domain and multiple highly conserved coiled-coil motifs.^3^, ^4^ TLK2 is known to have maximal activity during the S-phase of the cell cycle and is therefore tightly linked to DNA replication.^3^ DNA double-strand breaks lead to rapid and transient inhibition of TLK activity, suggesting a role in checkpoint regulation.^5^ With the discovery of both H3-H4 chaperone Asf1 and histone H3 as physiological substrates of TLKs, its protein function has been linked to chromatin assembly.^6^, ^7^, ^8^, ^9^, ^10^

To establish the contribution of *TLK2* variants to NDDs in humans, we systematically collected phenotypic data of the five affected individuals with *TLK2* variants reported previously,^1^ derived cell lines, and exploited different strategies to identify additional individuals with a variant in this gene. By including *TLK2* in a Deciphering Developmental Disorders^11^ Complementary Analysis Project, by using of GeneMatcher,^12^ and by sharing data with international collaborators, we identified a total of 38 unrelated individuals and two affected mothers with heterozygous variants in *TLK2*. Variants were detected by either family-based WES (research settings, n = 18 probands and 2 affected parents; diagnostic settings, n = 18 probands) or whole-genome sequencing (WGS) (research settings, n = 2 probands) in 26 different institutions and 7 different countries (Figure S1; Supplemental Subjects and Methods). Two additional individuals with *de novo TLK2* variants c.1514T>A (p.Val505Asp) and c.2171G>A (p.Arg724Gln), each of whom had a second likely pathogenic mutation in another gene, were excluded from further consideration to avoid confounding in the phenotypic analysis (Supplemental Subjects and Methods). IRB-approved consents for WES or WGS in diagnostic or research settings were obtained for all individuals.

**Figure 1:**
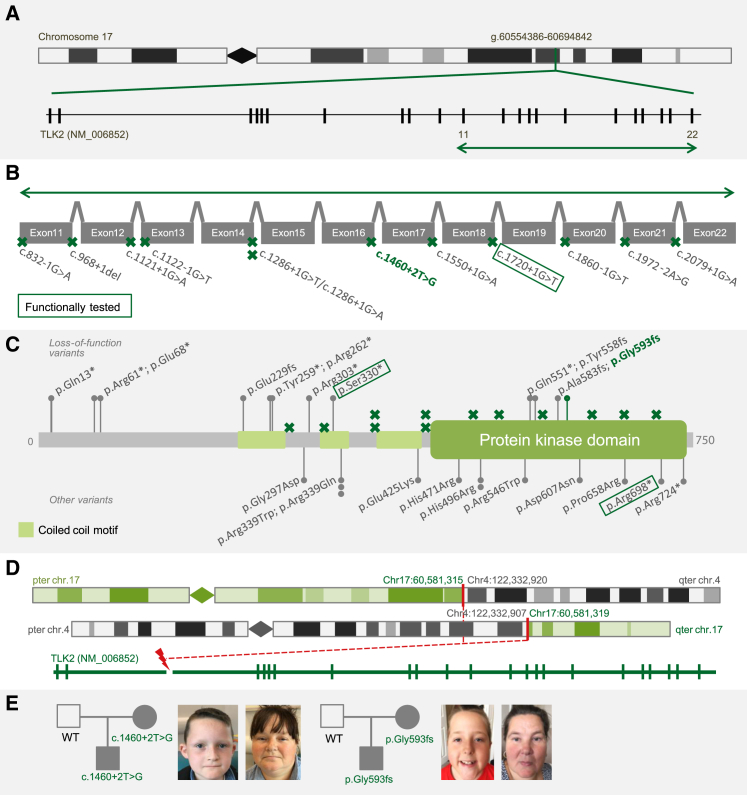
Intragenic Variants and Balanced Translocation Identified in *TLK2* (A) Location of *TLK2* (GenBank: NM_006852.3) on chromosome 17q23.2 (see Supplemental Subjects and Methods for discussion about different *TLK2* spliceforms). Vertical marks in *TLK2* represent the 22 exons. Green arrow indicates region enlarged in panel below. (B) Schematic view (not to scale) of exons 11–22 and locations of 12 identified splice site mutations (green crosses). The splice site mutation inherited from an affected parent is shown in bold and green. The variant subjected to cDNA analysis is shown in the dark green rectangle. (C) Overview of TLK2 protein with the protein kinase domain (dark green) and three coiled-coil motifs (light green). Loss-of-function variants (24 total, including 8 nonsense, 4 frameshift, and 12 splice site mutations) are shown above the protein with green crosses indicating positions of splice site mutations. Other variants (11 missense variants and 2 nonsense variants causing a premature stop codon in the last exon) are shown below the protein. The frameshift mutation inherited from an affected parent is shown in bold and green. The variants subjected to cDNA analysis are shown in the dark green rectangles. (D) Balanced translocation between chromosomes 4 and 17, with the breakpoint disrupting *TLK2* between exons 2 and 3, identified in one individual: 46,XX,t(4;17)(27;q23.2).seq[GRCh37]t(4;17)g.[chr4:pter_cen_122332907:: chr17:60,581,319_qter]g.[chr17_pter_cen_60,581,315::chr4:122,332,920_qter]. (E) Pedigrees of individuals with inherited variants and photographs of probands and their affected mothers. Both mothers have facial dysmorphism similar to their children. WT, wild-type at variant position.

We observed a broad spectrum of different variant types in *TLK2* (GenBank: NM_006852): 4 frameshift variants, 10 nonsense variants (including 2 located in the last exon), 12 canonical splice-site variants, and 9 missense variants (Figures 1A–1C; Table 1). Additionally, we identified a *de novo* balanced translocation in one of the WGS case subjects, resulting in a breakpoint at chromosome 17q23.2 disrupting the *TLK2* intron between exons 2 and 3 (Figure 1D; Supplemental Subjects and Methods). Interestingly, we found recurrent mutations within our cohort of affected individuals, occurring at hypermutable sites as reported by Rahbari et al.^13^ We considered the alternative possibility of gene conversion, because pseudogenes very similar to *TLK2* exist at 10p11.21 and/or 17q12; however, the pseudogene sequence(s) at the site of each recurrent mutation correspond to wild-type *TLK2*, excluding this mechanism. The missense variants c.1487A>G (p.His496Arg) and c.1015C>T (p.Arg339Trp) were each identified in two unrelated individuals, and c.1016G>A (p.Arg339Gln) also occurs at the Arg339 codon (Figure 1C; Table 1). Finally, two splice variants were predicted to give rise to the same affected protein product: c.1286+1G>T and c.1286+1G>A (Figure 1B; Table 1). From the 9 missense variants identified in 11 unrelated individuals, 5 are located in the catalytic domain of the protein and 3 in a coiled-coil motif. One variant, c.890G>A (p.Gly297Asp), is located outside a known functional domain, but affects a highly conserved amino acid and was predicted pathogenic by several *in silico* prediction programs, similar to other missense variants (Figure 1C; Table S1). None of the missense variants were present in the ExAC database,^14^ nor in our in-house database of variants identified in healthy control subjects. The recently released gnomAD database, containing WGS variants identified in control subjects, reported only c.1636C>T (p.Arg546Trp) in a single individual (allele frequency of ∼0.000004). None of the other missense variants were present in the gnomAD database (Table 1).

**Table 1:** Intragenic Variants in *TLK2* (GenBank: NM_006852.3), Inheritance, and Presence in ExAC and gnomAD Databases

**Subgroup**	**cDNA Position**	**Protein Position**	**Inheritance**	**RNA Analysis**	**cMAF ExAC**	**cMAF gnomAD**
Predicted LOF	c.37C>T	p.Gln13^∗^	*de novo*	no	no LOF variants	5 LOF variants: ∼0.00002
	c.181C>T	p.Arg61^∗^	*de novo*	no		
	c.202G>T	p.Glu68^∗^	*de novo*	no		
	c.685_688del	p.Glu229Argfs^∗^6	*de novo*	no		
	c.777C>A	p.Tyr259^∗^	*de novo*	no		
	c.784C>T	p.Arg262^∗^	*de novo*	no		
	c.832−1G>A	unknown	*de novo*	no		
	c.907C>T	p.Arg303^∗^	*de novo*	no		
	c.968+1del	unknown	*de novo*	no		
	c.989C>A	p.Ser330^∗^	*de novo*	yes		
	c.1121+1G>A	unknown	*de novo*	no		
	c.1122−1G>T	unknown	*de novo*	no		
	c.1286+1G>T	unknown	*de novo*	no		
	c.1286+1G>A	unknown	*de novo*	no		
	c.1460+2T>G	unknown	inherited	no		
	c.1550+1G>A	unknown	*de novo*	no		
	c.1651C>T	p.Gln551^∗^	*de novo*	no		
	c.1672dup	p.Tyr558Leufs^∗^4	*de novo*	no		
	c.1720+1G>T^a^	unknown	*de novo*	yes		
	c.1746delA	p.Ala583Argfs^∗^5	*de novo*	no		
	c.1776_1783delTGGTCTTT	p.Gly593Glufs^∗^5	inherited	no		
	c.1860−1G>T	unknown	unknown	no		
	c.1972−2A>G	unknown	*de novo*	no		
	c.2079+1G>A	unknown	*de novo*	no		
Other variant types	c.2092C>T^a^	p.Arg698^∗^	*de novo*	yes	0	0
	c.2170C>T	p.Arg724^∗^	*de novo*	no	0	0
	c.890G>A	p.Gly297Asp	*de novo*	no	0	0
	c.1015C>T	p.Arg339Trp	*de novo*^b^	no	0	0
	c.1016G>A	p.Arg339Gln	*de novo*	no	0	0
	c.1273G>A	p.Glu425Lys	unknown	no	0	0
	c.1412A>G^a^	p.His471Arg	*de novo*	no	0	0
	c.1487A>G^a^	p.His496Arg	*de novo*^b^	no	0	0
	c.1636C>T	p.Arg546Trp	*de novo*	no	0	∼0.000004
	c.1819G>A^a^	p.Asp607Asn	*de novo*	no	0	0
	c.1973C>G	p.Pro658Arg	*de novo*	no	0	0

Identified balanced translocation (n = 1) is not included in this table. Abbreviations: cMAF, cumulative minor allele frequency; LOF, loss-of-function

a Variant reported previously^1^

b Recurrent *de novo* variant identified in two unrelated individuals

For all but two variants (Table 1), the *de novo* status was assessed by sequencing the parents of the proband. In two individuals, variants were inherited from a similarly affected parent, while all other variants (n = 34) occurred *de novo*. Detailed phenotyping revealed that both mothers carrying a predicted loss-of-function (LOF) *TLK2* variant (Table 1) were mildly affected. The first mother (c.1460+2T>G) had mild neurodevelopmental delay and speech delay. The second affected mother (c.1776_1783delTGGTCTTT [p.Gly593Glufs^∗^5]) had a low-normal IQ level but was diagnosed with bipolar disorder. Both had facial dysmorphism similar to their affected children (Figure 1E). The inherited variants illustrate that the search for a diagnosis should not always be restricted to *de novo* mutations, in particular if individuals are only mildly affected. Similar to the parents in this study, who were never referred for genetic testing before investigation of their child uncovered a *TLK2* variant, we expect mutations causing milder phenotypes to be present in the general population. This could explain why, although *TLK2* exhibits very strong constraint against LOF variants (pLI = 1), five LOF variants (low-coverage variants excluded) have been reported in gnomAD, and a missense variant—c.1636C>T (p.Arg546Trp)—that was reported here as *de novo* variant, was present at very low allele frequency in the population (aggregate minor allele frequency of LOF and missense variants ∼0.000024).

**Figure 2:**
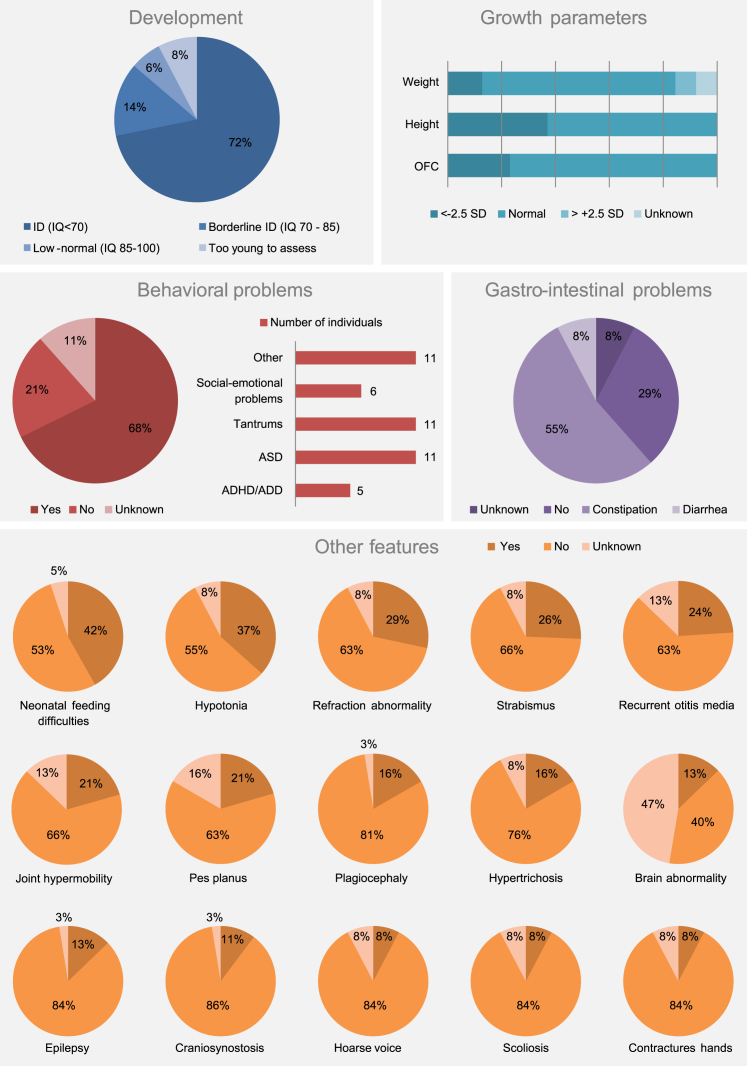
Clinical Spectrum Associated with *TLK2* Variants Overview of clinical features observed in individuals with *TLK2* variants.

**Figure 3:**
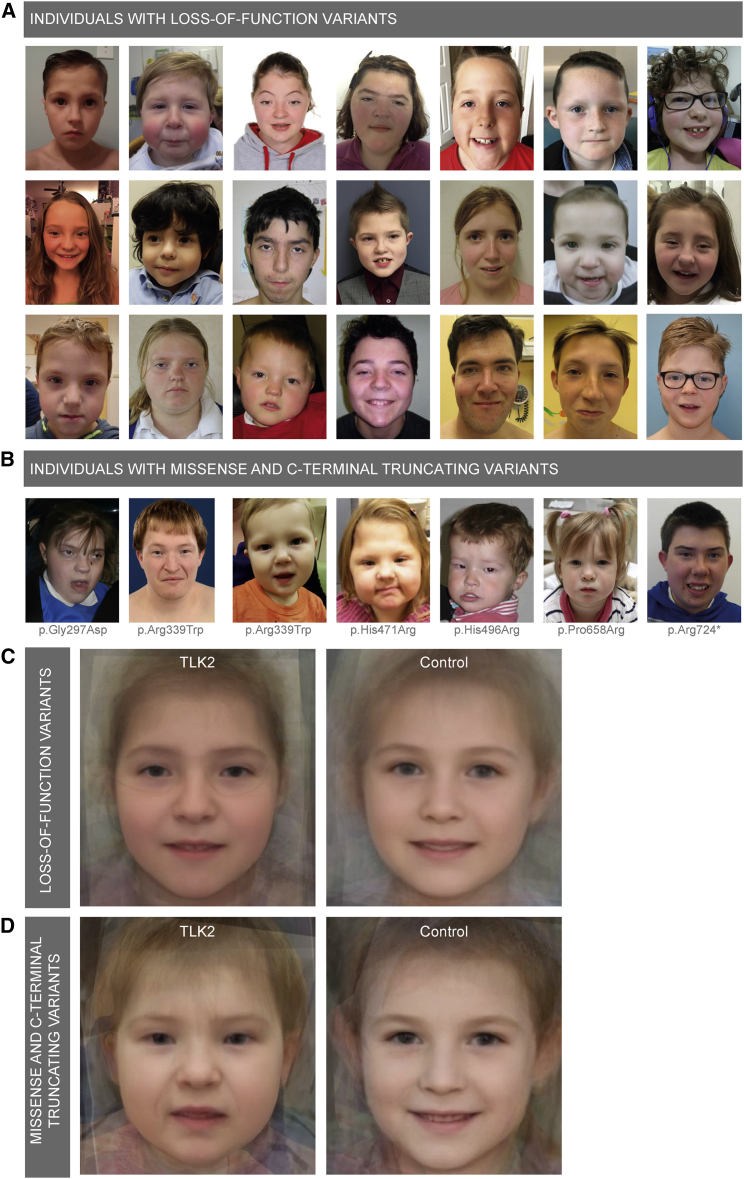
Facial Dysmorphism of Individuals with *TLK2* Variants (A) Photographs of 21 unrelated individuals with a loss-of-function variant in *TLK2*, showing overlapping facial dysmorphism. Most frequently reported by clinicians were blepharophimosis, telecanthus, prominent nasal bridge, broad nasal tip, thin vermilion upper lip, and upward slanted palpebral fissures. Pointed and tall chin, epicanthal folds, narrow mouth, high palate, microtia, posteriorly rotated ears, long face, ptosis, and asymmetric face were observed in fewer than half of the individuals. (B) Photographs of seven unrelated individuals with a missense or C-terminal truncating variant in TLK2. Variant c.2170C>T (p.Arg724^∗^) is assigned to this subgroup, since a premature stop codon is introduced in the last exon. Facial dysmorphisms overlapped with dysmorphism observed in individuals with loss-of-function variants. (C) Computational averaging of 33 facial photographs of 22 subjects with LOF variants in *TLK2* (left) compared with 22 gender- and age-matched control subjects (right). (D) Computational averaging of 11 facial photographs of 8 subjects with missense or C-terminal truncating variants in *TLK2* (left) compared with 8 gender- and age-matched control subjects (right).

Consistent with the phenotypes of both affected mothers, mild neurodevelopmental phenotypes accompanied by language and motor delay were present in the majority of the 38 unrelated probands: 6% of the individuals had normal IQ levels (85–100), 14% had borderline ID (IQ 70–85), and from the 72% diagnosed with ID (IQ < 70), most had mild ID (IQ 50–70) (Figure 2). Most of the affected probands (22 males and 16 females) were children at the time of last examination (median 8.0 years; interquartile range 4.1–13.5 years); ages ranged between 3 months and 29 years. Three individuals, who all had language and motor delay, were too young for formal assessment of their neurodevelopmental phenotype. In addition to this, systematic evaluation of other clinical data, scored by the referring clinician, showed a variety of overlapping features (Figure 2, Table S2). Neurological problems including hypotonia (37%), epilepsy (13%), and non-specific intracranial brain abnormalities (13%) (Table S3) were observed. A broad range of behavioral disorders was present (68%), with often severely affected social functioning: tantrums (11 individuals), autism spectrum disorder (ASD; 11 individuals), attention-deficit disorder with or without hyperactivity (ADHD; 5 individuals), and severe social-emotional problems (6 individuals) were the most commonly reported problems. Less frequently observed were short attention span, pica disorder, aggression, obsessive-compulsive disorder, and anxiety in 11 individuals. Other recurrent features included gastro-intestinal problems (constipation in 55%; severe diarrhea in 8%), neonatal feeding difficulties (42%), eye abnormalities (refraction abnormality in 29%, strabismus in 26%), musculoskeletal abnormalities (joint hypermobility in 21%; pes planus in 21%; toe walking in 18%; scoliosis in 8%; contractures of the hands in 8%), recurrent otitis media (24%), hypertrichosis (16%), and hoarse voice (8%). Abnormalities of skull shape were observed in 31% of probands (Figure 2, Tables S2 and S4), with clinically proven craniosynostosis being present in four (11%) of them (Table S5). However, sequence-based screening of 309 DNA samples from individuals with mixed, genetically undiagnosed craniosynostosis (Supplemental Subjects and Methods, Table S6) did not identify further case subjects, indicating that *TLK2* mutations are a rare cause of craniosynostosis. Growth parameters were frequently abnormal (Figure 2). Short stature was documented in 37%, microcephaly in 24% (primary in 13%, secondary in 3%, and unknown age of onset in 8%), and low body weight in 13%. Three individuals (8%) were overweight, with age of onset between the ages of 2 and 12 years. Features reported in only one or two individuals are summarized in Table S4. In addition to the other clinical features, overlapping facial dysmorphisms were present (Figures 3A and 3B). Most frequently reported by clinicians were blepharophimosis (82%), telecanthus (74%), prominent nasal bridge (68%), broad nasal tip (66%), thin vermilion of the upper lip (62%), and upslanting palpebral fissures (55%). Pointed and tall chin (42%), epicanthal folds (42%), narrow mouth (32%), high palate (30%), microtia, first degree (29%), posteriorly rotated ears (29%), long face (27%), ptosis (21%), and asymmetric face (16%) were observed in fewer than half of the individuals.

**Figure 4:**
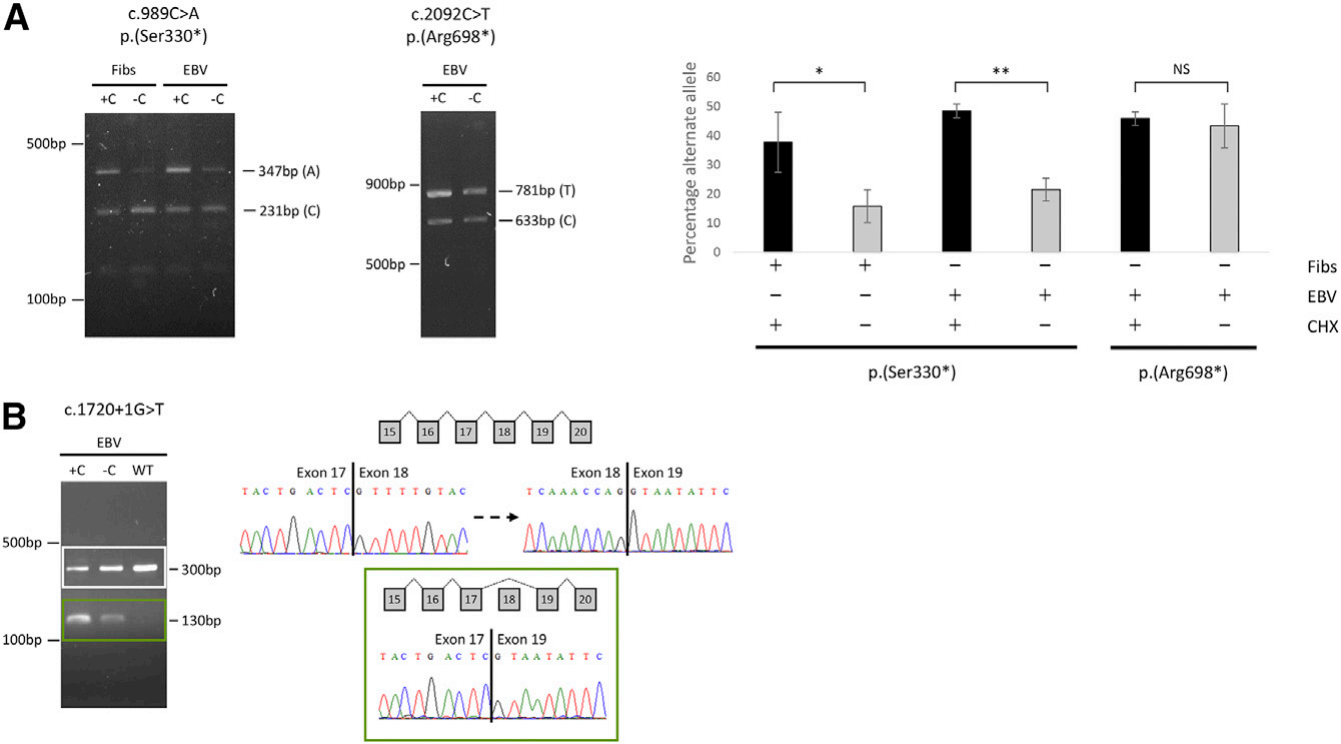
Analysis of *TLK2* Transcripts in Cell Lines (A) Analysis of transcripts encoding nonsense mutations c.989C>A (p.Ser330^∗^) and c.2092C>T (p.Arg698^∗^) in cell lines of affected individuals. Left panel shows reverse transcriptase-PCR (RT-PCR) products of cDNA prepared from fibroblast and lymphoblastoid cell lines of subject with p.Ser330^∗^ variant, either in the presence (+C) or absence (−C) of cycloheximide and incubated with ApoI (digests wild-type allele). Central panel shows RT-PCR of cDNA prepared from lymphoblastoid cell line of subject with p.Arg698^∗^ variant, in the presence (+C) or absence (−C) of cycloheximide and incubated with Hpy99I (digests wild-type allele). Right panel shows proportion (±standard deviation) of variant alleles quantified by deep sequencing of triplicate samples. Statistical testing of differences: ^∗^p = 0.046; ^∗∗^p = 0.011; NS, not significant. (B) Analysis of transcripts with canonical splice-site mutation c.1720+1G>T. A wild-type fragment at 300 bp in c.1720+1G>T lymphoblastoid cells is observed as well as a second fragment at 130 bp, which is absent in control cDNA. An increase of mutant transcript in cells was present when treated with cycloheximide (+C), indicating that the aberrant transcript was subject to NMD. Sequencing of the 300 bp (white box) and 130 bp (green box) fragments demonstrated skipping of exon 18 in the lower cDNA product. Abbreviations: Fibs, fibroblasts; EBV, lymphoblastoid cells; C/CHX, cycloheximide; WT, control cDNA.

Analysis of data from the ExAC database demonstrates that *TLK2* is extremely intolerant for LOF variants (pLI score = 1).^14^ In line with this observation, animal models with depletion of TLK2 have been reported to have severely disturbed cellular and developmental processes. *Drosophila* with complete LOF of TLK were associated with arrested nuclear divisions, causing apoptosis of the cell.^7^ *Tlk2*-null mice were embryonically lethal due to placental failure.^15^ In this study, we found several predicted LOF variants in affected individuals. To investigate whether variants resulted in an aberrant transcript, we synthesized cDNA from RNA extracted from fibroblast or lymphoblastoid cell lines (Supplemental Subjects and Methods, Table S7) from three individuals with different variants: (1) c.989C>A (p.Ser330^∗^), predicted to result in a truncated product leading to nonsense-mediated decay (NMD); (2) c.2092C>T (p.Arg698^∗^), with a premature stop codon in the last exon predicted to escape from NMD; and (3) c.1720+1G>T, a mutation predicted to affect splicing of exon 18. To investigate the significance of NMD for expression of *TLK2* transcripts, we treated fibroblasts (for p.Ser330^∗^) and lymphoblastoid cell lines (for p.Ser330^∗^, p.Arg698^∗^, and c.1720+1G>T) with cycloheximide, an inhibitor of NMD.^16^ Transcript stability of cDNA PCR products from p.Ser330^∗^ and p.Arg698^∗^ individuals in the presence of cycloheximide was analyzed using a restriction enzyme assay targeting the wild-type transcript and the results were confirmed using deep sequencing to quantify relative levels of wild-type and mutant transcripts (Supplemental Subjects and Methods). For fibroblast and lymphoblastoid cell lines heterozygous for the p.Ser330^∗^ variant, the mutant allele represented 15.8% and 21.5% of transcripts, respectively, in the absence of cycloheximide, but rose to 37.7% and 48.5%, respectively, in the presence of cycloheximide, supporting that this variant is subject to NMD and causes haploinsufficiency of TLK2. In contrast, wild-type and mutant transcripts from lymphoblastoid cells of the individual heterozygous for p.Arg698^∗^ did not show significant differences between treated and untreated cells, supporting that the mutant transcript escapes NMD due to its location within the last coding exon of *TLK2* (Figure 4A). Amplification of cDNA from an individual with a splice-site variant (c.1720+1G>T) showed a full-length wild-type product of 300 bp and an additional aberrant smaller product of 130 bp, consistent with skipping of exon 18. Direct sequencing of this smaller fragment confirmed that exon 17 spliced directly to exon 19, thereby producing an out-of-frame transcript predicted to introduce a premature stop codon at the next amino acid position (p.Ser517fs^∗^1). Additionally, the intensity of the spliced transcript increased when treated with cycloheximide, indicating that the mutant transcript is subjected to NMD (Figure 4B).

By analyzing *TLK2* transcripts in cell lines of three different individuals, we were able to confirm that transcripts were subjected to NMD in two of them, causing haploinsufficiency of TLK2. It is likely that comparable variants predicted to cause LOF of TLK2 affect the transcript similarly. The large number of identified individuals with *TLK2* variants allowed us to search for underlying pathogenic mechanisms for the individuals with variants with unknown effect, such as p.Arg698^∗^. To assess this, we divided our cohort in two subgroups and (1) performed a structured genotype-phenotype analysis and (2) created and compared computationally modeled faces. Subgroup 1 (n = 25) included all probands carrying a predicted LOF variant (nonsense, frameshift or canonical splice-site, or balanced translocation) similar to variants p.Ser330^∗^ and c.1720+1G>T. Subgroup 2 (n = 13) comprised individuals with either missense variants or variants introducing a premature stop codon in the last exon of *TLK2*, such as p.Arg698^∗^. Affected parents of probands with inherited mutations were not included in the subgroups. Next, we compared frequencies of 40 different features and frequencies of 15 facial dysmorphisms between the two groups via a two-tailed Fisher’s exact test. This showed that both clinical features and facial dysmorphisms were remarkably similar between the two subgroups. From the 55 different features, none differed significantly between the two subgroups (p < 0.05), even without correction for multiple testing (Table S2). Second, averaged visualization of facial dysmorphism by computational modeling of 33 photographs from 22 individuals in subgroup 1 compared with 11 photographs from 8 individuals in subgroup 2 at different ages (Supplemental Subjects and Methods) showed consistent differences from a comparable number of gender- and age-matched controls, including blepharophimosis, telecanthus, broad nasal tip, and tall, pointed chin (Figures 3C and 3D). Given this strong overlap in phenotypes and facial dysmorphic features between probands with different type of mutations, it is likely that not only LOF variants but also the majority of identified missense variants and variants with a premature stop codon in the last exon have only a single functional copy of *TLK2*. Hence, we conclude that the predominant pathogenic mechanism of these *TLK2* mutations is haploinsufficiency.

Often mentioned together with TLK2 is its close interactor TLK1. From birth, murine *Tlk2* shows a similar expression pattern to the closely related paralog *Tlk1* across many tissues.^15^ Human TLK1 has 84% identity to TLK2 at the protein level,^3^ and it was shown that TLK1 depletion leads to extensive chromosome segregation defects in human cells.^17^ Interestingly, *TLK1* (MIM: 608438) is (similarly to *TLK2*) intolerant for both missense and truncating mutations in healthy individuals (significant z-scores of 3.84 [*TLK1*] and 5.67 [*TLK2*] and pLI [constraint] scores of 1.00 for both TLK1 and TLK2) (ExAC database).^14^ In the literature, four *de novo* variants have been reported in *TLK1* (GenBank: NM_012290.4): c.74C>T (p.Pro25Leu) in an individual with intellectual disability,^1^ c.1697T>C (p.Met566Thr) in an individual with autism,^18^ c.1796C>G (p.Ala599Gly) in an individual with a NDD and congenital heart disease,^19^ and c.1101del (p.Lys367Asnfs^∗^25) in an individual with schizophrenia.^20^ Importantly, none of these variants are present in the ExAC or gnomAD databases. Taking this into account, it is possible that *TLK1* variants could contribute to NDDs, similar to the homolog *TLK2*. In future research, the exact role of *TLK1* in NDDs should be further explored.

In conclusion, we show that both *de novo* and inherited mutations in *TLK2* cause a distinct neurodevelopmental disorder, hallmarked by mild developmental delay, a variety of behavioral disorders, severe gastro-intestinal problems, and facial dysmorphism. The identification of a large number of individuals (n = 40, including two affected mothers) emphasizes the power and importance of data sharing, allowing us to delineate the clinical phenotype and to evaluate genotype-phenotype correlations. More than two-thirds of the individuals were identified in two relatively small countries: the Netherlands and the UK (Figure S1). With an estimated prevalence of ∼1/566 (17/9,625) of *TLK2* variants in probands recruited to the DDD study, it is expected that a larger number of individuals with *TLK2* variants is present world-wide. In future, even more extensive data sharing than performed in this study will be needed to further extend the *TLK2* cohort. By analyzing three cell lines of affected individuals, we were able to confirm that at least two variants act through a heterozygous loss-of-function mechanism (haploinsufficiency). The phenotypes of these individuals and others with comparable loss-of-function variants significantly overlapped with phenotypes of individuals with other variant types, providing further evidence for the underlying disease mechanism of the *TLK2* variants. Given the genetic and functional similarities between TLK2 and TLK1, further research should focus on the potential role of *TLK1* mutations in developmental disorders.

## Declaration of Interests

The authors declare no competing interests.

## Web Resources

DECIPHER, https://decipher.sanger.ac.uk/
ExAC Browser, v.0.3.1, http://exac.broadinstitute.org/
GenBank, https://www.ncbi.nlm.nih.gov/genbank/
GeneMatcher, https://genematcher.org/
gnomAD Browser, v.r2.0.2, http://gnomad.broadinstitute.org/
OMIM, http://www.omim.org/

## Supplemental Data

Document S1. Figure S1, Tables S1–S7, and Supplemental Subjects and Methods

Document S2. Article plus Supplemental Data

## References

[1] Lelieveld S.H., Reijnders M.R., Pfundt R., Yntema H.G., Kamsteeg E.J., de Vries P., de Vries B.B., Willemsen M.H., Kleefstra T., Löhner K. (2016). Meta-analysis of 2,104 trios provides support for 10 new genes for intellectual disability. Nat. Neurosci..

[2] American Psychiatric Association (2013). Diagnostic and Statistical Manual of Mental Disorders, Fifth Edition (Washington, DC).

[3] Silljé H.H., Takahashi K., Tanaka K., Van Houwe G., Nigg E.A. (1999). Mammalian homologues of the plant Tousled gene code for cell-cycle-regulated kinases with maximal activities linked to ongoing DNA replication. EMBO J..

[4] Yamakawa A., Kameoka Y., Hashimoto K., Yoshitake Y., Nishikawa K., Tanihara K., Date T. (1997). cDNA cloning and chromosomal mapping of genes encoding novel protein kinases termed PKU-alpha and PKU-beta, which have nuclear localization signal. Gene.

[5] Groth A., Lukas J., Nigg E.A., Silljé H.H., Wernstedt C., Bartek J., Hansen K. (2003). Human Tousled like kinases are targeted by an ATM- and Chk1-dependent DNA damage checkpoint. EMBO J..

[6] Silljé H.H., Nigg E.A. (2001). Identification of human Asf1 chromatin assembly factors as substrates of Tousled-like kinases. Curr. Biol..

[7] Carrera P., Moshkin Y.M., Gronke S., Sillje H.H., Nigg E.A., Jackle H., Karch F. (2003). Tousled-like kinase functions with the chromatin assembly pathway regulating nuclear divisions. Genes Dev..

[8] Li Y., DeFatta R., Anthony C., Sunavala G., De Benedetti A. (2001). A translationally regulated Tousled kinase phosphorylates histone H3 and confers radioresistance when overexpressed. Oncogene.

[9] Klimovskaia I.M., Young C., Strømme C.B., Menard P., Jasencakova Z., Mejlvang J., Ask K., Ploug M., Nielsen M.L., Jensen O.N., Groth A. (2014). Tousled-like kinases phosphorylate Asf1 to promote histone supply during DNA replication. Nat. Commun..

[10] Bruinsma W., van den Berg J., Aprelia M., Medema R.H. (2016). Tousled-like kinase 2 regulates recovery from a DNA damage-induced G2 arrest. EMBO Rep..

[11] Deciphering Developmental Disorders Study (2017). Prevalence and architecture of *de novo* mutations in developmental disorders. Nature.

[12] Sobreira N., Schiettecatte F., Valle D., Hamosh A. (2015). GeneMatcher: a matching tool for connecting investigators with an interest in the same gene. Hum. Mutat..

[13] Rahbari R., Wuster A., Lindsay S.J., Hardwick R.J., Alexandrov L.B., Turki S.A., Dominiczak A., Morris A., Porteous D., Smith B., UK10K Consortium (2016). Timing, rates and spectra of human germline mutation. Nat. Genet..

[14] Lek M., Karczewski K.J., Minikel E.V., Samocha K.E., Banks E., Fennell T., O’Donnell-Luria A.H., Ware J.S., Hill A.J., Cummings B.B., Exome Aggregation Consortium (2016). Analysis of protein-coding genetic variation in 60,706 humans. Nature.

[15] Segura-Bayona S., Knobel P.A., González-Burón H., Youssef S.A., Peña-Blanco A., Coyaud É., López-Rovira T., Rein K., Palenzuela L., Colombelli J. (2017). Differential requirements for Tousled-like kinases 1 and 2 in mammalian development. Cell Death Differ..

[16] Ishigaki Y., Li X., Serin G., Maquat L.E. (2001). Evidence for a pioneer round of mRNA translation: mRNAs subject to nonsense-mediated decay in mammalian cells are bound by CBP80 and CBP20. Cell.

[17] Hashimoto M., Matsui T., Iwabuchi K., Date T. (2008). PKU-beta/TLK1 regulates myosin II activities, and is required for accurate equaled chromosome segregation. Mutat. Res..

[18] De Rubeis S., He X., Goldberg A.P., Poultney C.S., Samocha K., Cicek A.E., Kou Y., Liu L., Fromer M., Walker S., DDD Study, Homozygosity Mapping Collaborative for Autism, UK10K Consortium (2014). Synaptic, transcriptional and chromatin genes disrupted in autism. Nature.

[19] Homsy J., Zaidi S., Shen Y., Ware J.S., Samocha K.E., Karczewski K.J., DePalma S.R., McKean D., Wakimoto H., Gorham J. (2015). De novo mutations in congenital heart disease with neurodevelopmental and other congenital anomalies. Science.

[20] Fromer M., Pocklington A.J., Kavanagh D.H., Williams H.J., Dwyer S., Gormley P., Georgieva L., Rees E., Palta P., Ruderfer D.M. (2014). De novo mutations in schizophrenia implicate synaptic networks. Nature.

