# Supplementary material for: De Novo and Inherited Loss-of-Function Variants in TLK2: Clinical and Genotype-Phenotype Evaluation of a Distinct Neurodevelopmental Disorder
Source: Am J Hum Genet. 2018 May 31;102(6):1195–203. doi: 10.1016/j.ajhg.2018.04.014 (PMC5992133; doi:10.1016/j.ajhg.2018.04.014)
Supplement: Document S1. Figure S1, Tables S1–S7, and Supplemental Subjects and Methods [file mmc1.pdf]

The American Journal of Human Genetics, Volume *102*

## Supplemental Data

# *De Novo* and Inherited Loss-of-Function Variants in *TLK2*: Clinical and Genotype-Phenotype Evaluation of a Distinct Neurodevelopmental Disorder

**Margot R.F. Reijnders, Kerry A. Miller, Mohsan Alvi, Jacqueline A.C. Goos, Melissa M. Lees, Anna de Burca, Alex Henderson, Alison Kraus, Barbara Mikat, Bert B.A. de Vries, Bertrand Isidor, Bronwyn Kerr, Carlo Marcelis, Caroline Schluth-Bolard, Charu Deshpande, Claudia A.L. Ruivenkamp, Dagmar Wieczorek, The Deciphering Developmental Disorders Study, Diana Baralle, Edward M. Blair, Hartmut Engels, Hermann-Josef Lüdecke, Jacqueline Eason, Gijs W.E. Santen, Jill Clayton-Smith, Kate Chandler, Katrina Tatton-Brown, Katelyn Payne, Katherine Helbig, Kelly Radtke, Kimberly M. Nugent, Kirsten Cremer, Tim M. Strom, Lynne M. Bird, Margje Sinnema, Maria Bitner-Glindzicz, Marieke F. van Dooren, Marielle Alders, Marije Koopmans, Lauren Brick, Mariya Kozenko, Megan L. Harline, Merel Klaassens, Michelle Steinraths, Nicola S. Cooper, Patrick Edery, Patrick Yap, Paulien A. Terhal, Peter J. van der Spek, Phillis Lakeman, Rachel L. Taylor, Rebecca O. Littlejohn, Rolph Pfundt, Saadet Mercimek-Andrews, Alexander P.A. Stegmann, Sarina G. Kant, Scott McLean, Shelagh Joss, Sigrid M.A. Swagemakers, Sofia Douzgou, Steven A. Wall, Sébastien Küry, Eduardo Calpena, Nils Koelling, Simon J. McGowan, Stephen R.F. Twigg, Irene M.J. Mathijssen, Christoffer Nellaker, Han G. Brunner, and Andrew O.M. Wilkie**

**Figure S1: Overview of countries where *TLK2* mutations in probands have been identified.**

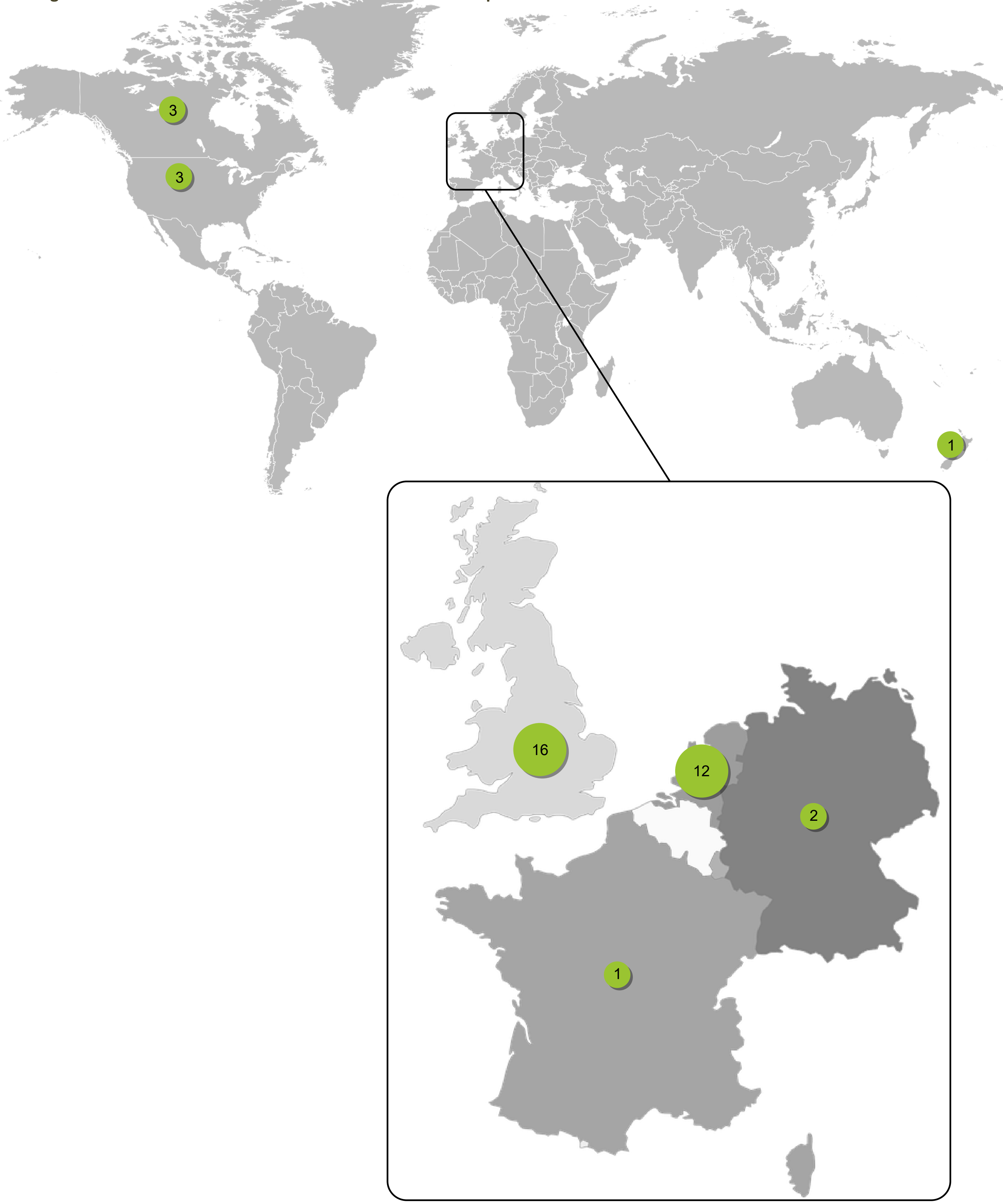

**Table S1.** Identified missense mutations in TLK2 (NM_006852) and predicted pathogenicity, domains, ExAC and gnomAD allele frequencies.

| cDNA change | Amino acid change | PhyloP | SIFT | Mutation Taster | Polyphen | Domain | ExAC allele frequency | gnomAD allele frequency |
|---|---|---|---|---|---|---|---|---|
| c.890G>A | p.(Gly297Asp) | 5.61 | 0.05 - Deleterious | Disease causing | 1.000 - Probably damaging | None | 0 | 0 |
| c.1015C>T[1] | p.(Arg339Trp) [1] | 6.18 | 0.04 - Deleterious | Disease causing | 0.210 - Benign | Coiled coil motif | 0 | 0 |
| c.1016G>A [1] | p.(Arg339Gln) [1] | 6.18 | 0.04 - Deleterious | Disease causing | 0.998 - Probably damaging | Coiled coil motif | 0 | 0 |
| c.1273G>A | p.(Glu425Lys) | 5.61 | 0.27 -Tolerated | Disease causing | 1.000 - Probably damaging | Coiled coil motif | 0 | 0 |
| c.1412A>G | p.(His471Arg) | 4.81 | 0.17 - Tolerated | Disease causing | 1.000 - Probably damaging | Catalytic domain | 0 | 0 |
| c.1487A>G [2] | p.(His496Arg)[2] | 4.73 | 0.12 - Tolerated | Disease causing | 0.995 - Probably damaging | Catalytic domain | 0 | 0 |
| c.1636C>T | p.(Arg546Trp) | 4.24 | 0 – Deleterious | Disease causing | 0.683 – Possibly damaging | Catalytic domain | 0 | 0.000004 |
| c.1819G>A | p.(Asp607Asn) | 6.02 | 0.65 – Tolerated | Disease causing | 0.008 – Benign | Catalytic domain | 0 | 0 |
| c.1973C>G | p.(Pro658Arg) | 6.02 | 0.65 – Tolerated | Disease causing | 1.000 - Probably damaging | Catalytic domain | 0 | 0 |

[1] Rows marked green indicate mutations affecting the same amino acid residue.
[2] Rows marked grey indicate recurrent mutations that were identified in two unrelated patients

**Table S2.** Frequencies of observed features and comparison between loss-of-function and other variant types.

| Feature | Loss-of-function mutations (%)* | Missense variants (%)* | Fisher's Exact test | Total frequency (%) |
|---|---|---|---|---|
| Short stature | 36 | 38 | p=1.000 | 37 |
| Low weight | 13 | 15 | p=1.000 | 14 |
| Overweight | 9 | 8 | p=1.000 | 8 |
| Microcephaly | 24 | 23 | p=1.000 | 24 |
| Intellectual disability (ID) | 70 | 83 | p=0.450 | 74 |
| Borderline ID | 23 | 0 | p=0.143 | 15 |
| Low-normal development | 5 | 9 | p=1.000 | 6 |
| Language delay | 92 | 92 | p=1.000 | 92 |
| Motor delay | 88 | 92 | p=1.000 | 89 |
| Behavior disorder | 77 | 75 | p=1.000 | 76 |
| Autism spectrum disorder | 23 | 50 | p=0.138 | 32 |
| Attention deficit - hyperactivity disorder (ADD/ADHD) | 18 | 8 | p=0.635 | 15 |
| Anxiety | 9 | 17 | p=0.602 | 12 |
| Tantrums | 27 | 42 | p=0.459 | 32 |
| Aggressiveness | 9 | 0 | p=0.529 | 6 |
| Obsessive compulsive disorder (OCD) | 9 | 0 | p=0.529 | 6 |
| Social-emotional problems | 18 | 17 | p=1.000 | 18 |
| Pica | 4 | 0 | p=1.000 | 3 |
| Short attention span | 4 | 8 | p=1.000 | 5 |
| Use of psychiatric drugs | 25 | 23 | p=1.000 | 24 |
| Hypotonia | 41 | 38 | p=1.000 | 40 |
| Epilepsy | 17 | 8 | p=0.638 | 14 |
| Brain abnormality | 27 | 20 | p=1.000 | 25 |
| Neonatal feeding difficulties | 43 | 46 | p=1.000 | 44 |
| Constipation | 55 | 69 | p=0.488 | 60 |
| Diarrhea | 9 | 8 | p=1.000 | 9 |
| Refraction abnormality | 33 | 27 | p=1.000 | 31 |
| Strabismus | 22 | 42 | p=0.258 | 29 |
| Other eye abnormality | 17 | 8 | p=0.646 | 14 |
| Craniosynostosis | 13 | 8 | p=1.000 | 11 |

| | | | | |
|---|---|---|---|---|
| Joint hypermobility | 18 | 36 | p=0.391 | 24 |
| Scoliosis | 9 | 8 | p=1.000 | 9 |
| Contractures hands | 9 | 8 | p=1.000 | 9 |
| Walking on tiptoes | 18 | 23 | p=1.000 | 20 |
| Pes planus | 24 | 27 | p=1.000 | 25 |
| Hoarse voice | 4 | 17 | p=0.266 | 9 |
| Plagiocephaly | 21 | 8 | p=0.394 | 16 |
| Recurrent otitis media | 30 | 20 | p=0.686 | 27 |
| Hypertrichosis | 23 | 8 | p=0.377 | 17 |
| Conductive hearing loss | 13 | 22 | p=0.602 | 16 |
| Sensorineural hearing loss | 8 | 0 | p=0.538 | 5 |
| **Facial dysmorphisms** | | | | |
| Asymmetric face | 20 | 8 | p=0.643 | 16 |
| Microtia | 36 | 15 | p=0.268 | 29 |
| Posteriorly rotated ears | 32 | 23 | P=0.714 | 29 |
| Long face | 29 | 25 | p=1.000 | 27 |
| Epicanthal folds | 31 | 60 | p=0.228 | 42 |
| Blepharophimosis | 80 | 85 | p=1.000 | 82 |
| Upward slanted palpebral fissures | 44 | 77 | p=0.086 | 55 |
| Ptosis | 24 | 15 | p=0.689 | 21 |
| Wide spaced eyes | 80 | 62 | p=0.263 | 74 |
| Prominent nasal bridge | 64 | 77 | p=0.486 | 68 |
| Broad nasal tip | 60 | 50 | p=0.473 | 66 |
| High palate | 21 | 50 | p=0.303 | 30 |
| Thin vermilion upper lip | 68 | 50 | p=0.470 | 62 |
| Narrow mouth | 24 | 50 | p=0.146 | 32 |
| Pointed, tall chin | 44 | 38 | p=1.000 | 42 |

* Frequencies in this table slightly differ from frequencies in the main text, since missing data (unknown presence of a feature) were excluded for statistical analysis. Clinical features of the two affected parents were not included in this analysis.

**Table S3.** Reported intracranial brain abnormalities.

| | **Description in MRI reports** |
|---|---|
| 1 | Dilated 3rd and 4th lateral ventricles |
| 2 | Tonsillar herniation; deep sella; severe scalloping; narrow peripheral CSF spaces; prominent CSF surrounding optic nerve |
| 3 | Slightly small anterior pituitary |
| 4 | Mild-moderate volume loss of cerebral white matter |
| 5 | Simple gyration pattern; lack of white matter |

**Table S4.** Overview of features reported in one or two patients.

| Feature | Number of patients |
|---|---|
| Delayed bone age | 1 |
| Anterior fontanel closed before age of 3 months | 1 |
| Brachycephaly, not related to craniosynostosis | 1 |
| Single testicle, hypoplastic scrotum, hypospadias | 1 |
| Hypoplastic nails | 1 |
| Immune deficiency (low IgG and IgM) | 1 |
| Cryptorchidism | 1 |
| Laryngomalacia | 1 |
| Restless legs | 1 |
| Persistent ductus arteriosus | 1 |
| Precocious puberty | 1 |
| Rheumatoid arthritis | 1 |
| Pigmentary variant of iris | 1 |
| Right supernumerary nipple | 1 |
| Bilateral uveo-retinal colobomata | 1 |
| Left cranial nerve IV palsy | 1 |
| Fibromyalgia | 1 |
| Right inguinal hernia | 1 |
| Sensorineural hearing loss with asymmetrical widened vestibular aqueducts or semicircular canals | 2 |

**Table S5.** Affected sutures in patients with craniosynostosis.

| | **Suture** |
|---|---|
| 1 | Bicoronal |
| 2 | Right coronal and sagittal |
| 3 | Metopic |
| 4 | Coronal and metopic |

**Table S6**. Primers used for the selective amplification of exons and flanking intron sequences from *TLK2.*

| ID | Target length (bp)* | Forward primer | Reverse primer | Multiplex PCR Mix |
|---|---|---|---|---|
| TLK2-Ex2 | 345 | ACACTGACGACATGGTTCTACAAATTACTGTGAGTTTTGTTCTACAG | TACGGTAGCAGAGACTTGGTCTACTATGTTAAATGACTACTGGAATGACC | 4 |
| TLK2-Ex3 | 284 | ACACTGACGACATGGTTCTACAACGCCATTGTATTCCAGCCGGGGTGAT | TACGGTAGCAGAGACTTGGTCTCAGCCTTGAGCCACCAAACCTGGCCCAAAC | 3 |
| TLK2-Ex4 | 401 | ACACTGACGACATGGTTCTACATAAGAGGAAGACAGTGATTCAGGAC | TACGGTAGCAGAGACTTGGTCTGAACTAACACTGTTCTGTCAGGTG | 1 |
| TLK2-Ex5 | 435 | ACACTGACGACATGGTTCTACATGGAGGAAATAGTCTGTTCTTG | TACGGTAGCAGAGACTTGGTCTATGTTGCCCAGGTTGGCCTCGAACT | 5 |
| TLK2-Ex6 | 337 | ACACTGACGACATGGTTCTACAGCATAGTACTGTTTTGAATTATTCATATCG | TACGGTAGCAGAGACTTGGTCTCTCTTCTGTAAAAAGCTAATTTACTGAC | 2 |
| TLK2-Ex7 | 314 | ACACTGACGACATGGTTCTACACTTATATTTGATAACTGTTTTTAACCCG | TACGGTAGCAGAGACTTGGTCTGAGCACTAGGGCAATGGAAAGGATA | 2 |
| TLK2-Ex8 | 415 | ACACTGACGACATGGTTCTACAGAACTTGGTATAAACCACCATGTCC | TACGGTAGCAGAGACTTGGTCTGTGGTCAGAGAAATACAGAGAAGTC | 2 |
| TLK2-Ex9 | 245 | ACACTGACGACATGGTTCTACAATTTGTGTGAGCAAGTGCTTTTTCC | TACGGTAGCAGAGACTTGGTCTGGTGCTTGCTATAAAATCTCTTACA | 4 |
| TLK2-Ex10 | 473 | ACACTGACGACATGGTTCTACAAAACATGCCCAAATTAGTAATTCAA | TACGGTAGCAGAGACTTGGTCTCAAATCATGTTCCTAAAAAGCTCTAC | 6 |
| TLK2-Ex11 | 368 | ACACTGACGACATGGTTCTACATTCTAAGAAGTGTCTTTATCCATGC | TACGGTAGCAGAGACTTGGTCTAGGACTTCACCTCATTCGATAC | 3 |
| TLK2-Ex12 | 325 | ACACTGACGACATGGTTCTACAAAATTGGATACACAAGTGACAAATTG | TACGGTAGCAGAGACTTGGTCTCTATTGCCGGTGACAATCAAC | 5 |
| TLK2-Ex13 | 392 | ACACTGACGACATGGTTCTACAGCTTTGAAGTTCTTCCCTCACATC | TACGGTAGCAGAGACTTGGTCTCACTGAAGCTTTCTGCTGCTATG | 5 |
| TLK2-Ex14 | 338 | ACACTGACGACATGGTTCTACATTACTGAACTCCTCTGTATGGTTTG | TACGGTAGCAGAGACTTGGTCTAGCAATCTCCAACCCAATATGC | 4 |
| TLK2-Ex15 | 451 | ACACTGACGACATGGTTCTACACTGGGAATTTTGCAAGCGTGG | TACGGTAGCAGAGACTTGGTCTTATGAGGCAGGAAGTACAGAACC | 2 |
| TLK2-Ex16 | 350 | ACACTGACGACATGGTTCTACATAATCACAAGTTTCAAGAAGGTGCT | TACGGTAGCAGAGACTTGGTCTACCAACAACAATGCACGTAAAG | 6 |
| TLK2-Ex17 | 409 | ACACTGACGACATGGTTCTACATCTCAATGGCTTGGTAGATTCC | TACGGTAGCAGAGACTTGGTCTTGTCACAAATTACTTGGTTCCCTC | 1 |
| TLK2-Ex18 | 421 | ACACTGACGACATGGTTCTACAAGGTAGTGTTAATCTGCTTGCTC | TACGGTAGCAGAGACTTGGTCTTCCAACACGCCCTCCTAAAC | 3 |
| TLK2-Ex19 | 329 | ACACTGACGACATGGTTCTACAAGTCCAGATTGCTTGATTCCC | TACGGTAGCAGAGACTTGGTCTGCCACATCTCTATAGCCAACCTG | 6 |
| TLK2-Ex20 | 368 | ACACTGACGACATGGTTCTACAGTACATGTCTTAAACTTATATGATC | TACGGTAGCAGAGACTTGGTCTCCTAGGGTTGAGGATTTCTGCT | - |
| TLK2-Ex21 | 431 | ACACTGACGACATGGTTCTACACCCACTCTGCTTTGACCTGGTAG | TACGGTAGCAGAGACTTGGTCTTTCACACTGAAGAATCCATCCA | 6 |
| TLK2-Ex22 | 452 | ACACTGACGACATGGTTCTACAAGAGGTACTTCTGTTGGTGCTT | TACGGTAGCAGAGACTTGGTCTGGATTCGCTATGTTCCAAACC | 1 |

*excluding universal adaptors CS1 and CS2 (underlined in the primer sequences; 22 nt each).

**Table S7.** Primers used for analysis of *TLK2* cDNA.

| *TLK2* mutation | Forward primer (5'→3') | Reverse primer (5'→3') | Target length (bp)* | Restriction enzyme |
|---|---|---|---|---|
| c.989C>A p.(S330*) | ACACTGACGACATGGTTCTACATGCAAGACCGCTTGAGACTG | TACGGTAGCAGAGACTTGGTCTCAGCTCTGCCTGGATCTCTG | 347 | ApoI |
| c.1720+1G>T | ACACTGACGACATGGTTCTACAGCATGCATGTAGGGAATACCG | TACGGTAGCAGAGACTTGGTCTATCTCTCCACACGCTGTACC | 300 | - |
| c.2092C>T p.(R698*) | ACACTGACGACATGGTTCTACAGCATGCATGTAGGGAATACCG | TACGGTAGCAGAGACTTGGTCTACTGTTATTGGACGCCCCAG | 781 | Hpy99I |

*excluding universal adaptors CS1 and CS2 (underlined in the primer sequences; 22 nt each)

**SUPPLEMENTAL METHODS**

## ***1. Identification of TLK2 variants***

**Overview**

| Method | Setting | Number of individuals |
|---|---|---|
| WES | Diagnostics | 18 probands |
| WES (A) | Research | 18 probands + 2 affected parents |
| WGS (B) | Research | 2 probands |

**A) Whole exome sequencing in research settings (*n*=18+2)**

Oxford study (*n*=1)

WES was performed as part of a study of seven unrelated individuals with bicoronal synostosis as described by Sharma *et al.*[1] Initial filtering, performed as described[1] did not highlight any strong candidate variant, but after comparison with WGS data from another craniosynostosis cohort (individual 2, WGS in research settings, see below), the *TLK2* c.989C>A, p.(S330*) variant was identified and prioritized for further analysis. The study has ethical approval from Oxfordshire Research Ethics Committee B (reference C02.143), and London Riverside Research Ethics Committee (reference 09/H0706/20).

DDD study (*n*=15 probands +2 affected parents)

Following identification of *TLK2* mutations in the Rotterdam and Oxford studies, rare *TLK2* variants were requested from the Deciphering Developmental Disorders (DDD) research study (http://www.ddduk.org)[2; 3] as part of the approved Complementary Analysis Project #144. Data were initially obtained from Datafreeze 2 and later extended to Datafreeze 3, comprising 7,833 trios and 1,792 singletons with undiagnosed developmental disorders, primarily developmental delay/ learning disability. Non-synonymous variants absent from ExAC in 17 unrelated probands were prioritized and the referring clinician contacted, requesting further clinical information and DNA for confirmatory dideoxy-sequencing. Two cases had a mutation inherited from an affected parent. Two further cases were excluded from the analysis, one because a co-existing nonsense mutation in *KIF11* confounded the phenotypic interpretation and the other because the parents could not be re-contacted. The study has UK Research Ethics Committee approval (10/H0305/83, granted by the Cambridge South REC, and GEN/284/12 granted by the Republic of Ireland REC).

MRBE study (*n*=2)

WES and filtering for rare *de novo* variants was performed as published previously by Schäfgen et al.[4] as part of a study on 311 individuals with ID / developmental delay (DD) with or without additional features (e.g. craniofacial dysmorphism, organ malformation etc.) that could not be attributed to a clinically recognisable syndrome by experienced clinical geneticists. All investigations were performed in accordance with the Declaration of Helsinki and were approved by the local institutional review boards (Ethics Committee of the Medical Faculty of the University of Bonn, approvals 131/08 and 024/12, Ethics Committee of the Medical Faculty of the University of Essen 08-3663).

<u>HUGODIMS study</u>

WES was performed as part of the research program conducted by the Western France consortium HUGODIMS (French acronym standing for "Projet inter-régional Français des Hôpitaux Universitaires du Grand Ouest pour l'exploration par approche exomique des causes moléculaires de Déficience Intellectuelle isolée ou syndromique Modérée à Sévère").[5] Two *de novo* candidate missense variants were highlighted, in *GABRA1* and *TLK2.* Although the *TLK2* variant is likely to contribute to the phenotype of this patient, a contribution of the *GABRA1* variant cannot be excluded. Therefore, this patient was excluded from further phenotypic analysis. All families gave written informed consent for inclusion in the study which has been approved by the CHU de Nantes-ethics committee (number CCTIRS: 14.556).

**B) Whole genome sequencing in research settings (*n*=2)**

<u>Individual 1: Breakpoint mapping of balanced translocation by WGS</u>

Standard RHG and GTG karyotype identified an apparently balanced reciprocal translocation 46,XX,t(4;17)(q27;q23). Array-CGH did not identify any pathogenic imbalances (Human Genome CGH microarray 180K, Agilent, Santa Clara, CA, USA). A whole genome library was prepared with 3 µg genomic blood DNA following the Illumina TruSeq protocol (Illumina, San Diego, California, USA). A 350 bp fragment library was sequenced on an Illumina NextSeq 500 as paired-end 101 bp reads. The sequencing depth was 8.71x. Alignment of the reads against the GRCh37 version of the human genome was done using BWA-MEM v 0.7.10.[6] The reads were then sorted using Samtools v 1.3.1[7], and the duplicates removed by PicardTools v 1.138 (picard.sourceforge.net). Then, the structural variants (SV) were detected using BreakDancer v 1.4.5[8] and annotated using an in-house script, mainly for the purpose of filtering them on the basis of their occurrence in a local database. Integrative Genomics Viewer v 2.3 was used for the SV visualization.[9]

In order to amplify junction fragments, primer pairs were selected on each side of the breakpoint region delimited by WGS (primers sequence available on request). PCR amplification was performed using the Taq DNA Core kit 25 (MP Biomedicals, Solon, Ohio). DNA from a non-carrier of the chromosomal rearrangement was amplified as a negative control. Specific PCR products corresponding to the junction fragment were sequenced by the dideoxy method.

<u>Individual 2: WGS</u>

WGS was performed on DNA of the proband and his parents by Complete Genomics, a BGI company (Mountain View, CA, USA) as described by Drmanac et al..[10] Variants were annotated using GRCh37/hg19 and dbSNP build 130. Data were analyzed using cga tools version 1.6.0.43 and TIBCO Spotfire 7.0.0 (TIBCO Software Inc., Boston, MA, USA). A *de novo* disease model was tested using the calldiff script (Python script kindly provided by Complete Genomics) as described by Gilissen et al.,[11] which identified 76 *de novo* high score variants (both somatic scores ≥5), with only one that was protein affecting (c.907C>T in *TLK2*). Also, *de novo* X-linked variants were excluded. The study has approval of the Medical Ethical Committee of the Erasmus University Medical Center Rotterdam (MEC-2012-140 and MEC-2013-547).

## ***<u>2. Confirmation of research variants</u>***

All variants identified in *TLK2* were numbered according to Genbank accession NM_006852.3 (ENST00000346027.9), a transcript comprising 22 exons that encodes a 750 amino acid protein. An alternative transcript containing an additional 66 nucleotide exon between exons 12 and 13 has been annotated (NM_001284333), but the additional exon is poorly conserved in mammalian species and its physiological significance is uncertain. Confirmation of genetic variants identified in *TLK2* by research exome or whole-genome sequencing was carried out by dideoxy-sequencing of genomic PCR amplification products. Amplification of products utilized primers shown in Table S1; sequencing and visualization of variants was performed as previously described.[12]

## ***<u>3. Screening of individuals with craniosynostosis</u>***

We screened all 22 coding exons including exon/intron boundaries of *TLK2* in a mixed cohort of craniosynostosis patients using primers optimized for amplification of *TLK2* only (to ensure no amplification of unprocessed or processed partial pseudogenes located on chromosomes 4, 7, 10 and 17). Primers were designed to amplify target regions between 245-473 bp with the addition of CS1 (5'-ACACTGACGACATGGTTCTACA-3') and CS2 (5'-TACGGTAGCAGAGACTTGGTCT-3') adaptor sequences included on the 5' ends of all target-specific forward and reverse primers, respectively. Briefly, primers were multiplexed into pools of 3-4 optimized pairs (with the exception of exon 20,

which was amplified independently), with a final concentration of 0.5 µM per primer in a reaction volume of 20 µl, containing 1x Buffer, 200 µM each dNTP and 0.5 U of Q5® High-Fidelity DNA Polymerase (New England BioLabs). Cycling conditions consisted of a 30 s denaturation step at 98°C, followed by 35 cycles of 98°C for 10 s, 60°C for 30 s and 72°C for 30 s, and a final extension step of 72°C for 10 min. Illumina-specific sequence adaptors and 10 bp sample indexes were attached using the Access Array™ Barcode Library for Illumina® Sequencers-384, Single Direction (Fluidigm) at a concentration of 0.4 µM per primer with Q5® High-Fidelity DNA Polymerase as above, for 9 cycles only. Primer/adaptor sequences and details of multiplex pools are provided in Table S1.

Indexed PCR products were pooled, purified with AxyPrep MAG PCR Clean-Up Kit (Axygen) and quantified using the 2200 TapeStation (Agilent Technologies) with a High Sensitivity D1000 ScreenTape and a Qubit® 1.0 Fluorometer (ThermoFisher Scientific), following the manufacturer's instructions. Pooled and indexed PCR products were diluted to a final concentration of 9 pM and sequenced using the Illumina MiSeq platform with a MiSeq Reagent Kit v2 for 500 cycles (Illumina) according to the manufacturer's instructions. All targets of *TLK2* were sequenced with a minimum coverage of 10 reads in a total of 309 samples from individuals with mixed types of craniosynostosis without a defined genetic cause.

## ***4. Analysis of TLK2 Splicing***

Skin fibroblasts from an individual heterozygous for the *TLK2* c.989C>A, p.(Ser330*) mutation were cultured in Dulbecco modified Eagle medium (DMEM) supplemented with 10% fetal bovine serum (FBS) and 1% penicillin and streptomycin. Lymphoblastoid cell lines were cultured in RPMI-1640 supplemented with 15% FBS and 1% penicillin and streptomycin. All reagents were purchased from Life Technologies unless otherwise stated. Patient cells were cultured in two concurrent T25 flasks, incubated with either 100 ng cycloheximide or 1% DMSO. Flasks were incubated at 37°C with 5% $CO_2$ for 4 h, then resuspended in Trizol for RNA extraction. Total RNA was extracted using the Direct-zol™ RNA MiniPrep Kit (Zymo Research) according to the manufacturer's instructions. gDNA was removed by treatment with DNase I (Sigma-Aldrich), following the manufacturer's protocol. cDNA was generated from 1 µg RNA with the addition of 0.1 µg random oligomers and 0.25 µg oligo dT primers using the RevertAid First Strand cDNA Synthesis kit (ThermoFisher Scientific). The reaction mixture was incubated at 65°C for 5 min, chilled on ice prior to addition of other reaction components, then incubated for 5 min at 25°C for 5 min followed by 60 min at 42°C before being terminated by heating to 70°C for 5 min. Primers used for RT-PCR and deep sequencing are shown in Table S2 (note, the CS1 and CS2 tags were omitted for agarose gel visualization but included for deep sequencing).

Transcripts were analyzed by gel electrophoresis of cDNA digested with a restriction enzyme targeting the wild-type transcript. The aberrant splice product obtained from the patient with c.1720+1G>T mutation was extracted using the High Pure PCR Product Purification Kit (Roche) and dideoxy-sequencing was performed as above. cDNA was amplified in triplicate for deep sequencing and indexed as above with a minimum coverage of 332 at the variant position.

***5. Bioinformatic analysis of deep sequencing data***

Variant calls, allele counts and coverage information were obtained with amplimap (https://github.com/koelling/amplimap), a pipeline built using *Snakemake* version 3.11.2.[13] Reads were trimmed to remove primers and aligned to GRCh37 (without alt contigs) using *bwa mem* version 0.7.12[14] for genomic DNA and *STAR* version 2.5.1b[15] for cDNA. For the allele balance analysis the two target SNPs were masked to prevent reference bias during mapping. Coverage was calculated using *bedtools* version 2.25.0[16], requiring a minimum coverage of 10 to consider a target region fully covered. Variants were called with *Platypus* version 0.8.1[17] and annotated using *Annovar* version 2015-06-17[18]. Further processing and annotation was performed in Python 3.5.3 with *pysam*[19], *biopython*[20] and *pandas.*[21]

***6. Computational analysis of facial photographs***

To visualise the characteristic facial features of groups of subjects, we generate realistic, de-identified average faces. We used a fully-automated algorithm that (1) annotates a face with a constellation of 68 facial feature points, (2) creates an average face mesh and (3) morphs the face of each subject onto the average face mesh. The face averaging algorithm was developed from previous work,[22; 23] and improved upon to produce more visually appealing, and better de-identified averages. We used an ensemble of regression trees to detect a constellation of 68 feature points on the face.[24] The face mesh of each patient was aligned to a target mesh, which was created from healthy control patients, using the feature points along the middle of the face. The average face was created by morphing the image of each patient’s face onto the average face mesh. To avoid biases towards individuals with multiple images, each patient's contribution to the average face mesh was equally weighted. Finally, to avoid variances in illumination between images, we normalized the pixel values within the face to an average value across all faces for each average.

**SUPPLEMENTAL REFERENCES**


1. Sharma, V.P., Fenwick, A.L., Brockop, M.S., McGowan, S.J., Goos, J.A., Hoogeboom, A.J., Brady, A.F., Jeelani, N.O., Lynch, S.A., Mulliken, J.B., et al. (2013). Mutations in TCF12, encoding a basic helix-loop-helix partner of TWIST1, are a frequent cause of coronal craniosynostosis. Nat Genet 45, 304-307.
2. Deciphering Developmental Disorders Study. (2015). Large-scale discovery of novel genetic causes of developmental disorders. Nature 519, 223-228.
3. Deciphering Developmental Disorders Study. (2017). Prevalence and architecture of de novo mutations in developmental disorders. Nature 542, 433-438.
4. Schafgen, J., Cremer, K., Becker, J., Wieland, T., Zink, A.M., Kim, S., Windheuser, I.C., Kreiss, M., Aretz, S., Strom, T.M., et al. (2016). De novo nonsense and frameshift variants of TCF20 in individuals with intellectual disability and postnatal overgrowth. Eur J Hum Genet 24, 1739-1745.
5. Isidor, B., Kury, S., Rosenfeld, J.A., Besnard, T., Schmitt, S., Joss, S., Davies, S.J., Lebel, R.R., Henderson, A., Schaaf, C.P., et al. (2016). De novo truncating mutations in the kinetochore-microtubules attachment gene CHAMP1 Cause Syndromic Intellectual Disability. Hum Mutat 37, 354-358.
6. Li, H. (2013) Aligning sequence reads, clone sequences and assembly contigs with BWA-MEM. arXiv preprint arXiv:13033997.
7. Li, H., Handsaker, B., Wysoker, A., Fennell, T., Ruan, J., Homer, N., Marth, G., Abecasis, G., Durbin, R., and Genome Project Data Processing, S. (2009). The Sequence Alignment/Map format and SAMtools. Bioinformatics 25, 2078-2079.
8. Chen, K., Wallis, J.W., McLellan, M.D., Larson, D.E., Kalicki, J.M., Pohl, C.S., McGrath, S.D., Wendl, M.C., Zhang, Q., Locke, D.P., et al. (2009). BreakDancer: an algorithm for high-resolution mapping of genomic structural variation. Nat Methods 6, 677-681.
9. Thorvaldsdottir, H., Robinson, J.T., and Mesirov, J.P. (2013). Integrative Genomics Viewer (IGV): high-performance genomics data visualization and exploration. Brief Bioinform 14, 178-192.
10. Drmanac, R., Sparks, A.B., Callow, M.J., Halpern, A.L., Burns, N.L., Kermani, B.G., Carnevali, P., Nazarenko, I., Nilsen, G.B., Yeung, G., et al. (2010). Human genome sequencing using unchained base reads on self-assembling DNA nanoarrays. Science 327, 78-81.
11. Gilissen, C., Hehir-Kwa, J.Y., Thung, D.T., van de Vorst, M., van Bon, B.W., Willemsen, M.H., Kwint, M., Janssen, I.M., Hoischen, A., Schenck, A., et al. (2014). Genome sequencing identifies major causes of severe intellectual disability. Nature 511, 344-347.
12. Miller, K.A., Twigg, S.R., McGowan, S.J., Phipps, J.M., Fenwick, A.L., Johnson, D., Wall, S.A., Noons, P., Rees, K.E., Tidey, E.A., et al. (2017). Diagnostic value of exome and whole genome sequencing in craniosynostosis. J Med Genet 54, 260-268.
13. Koster, J., and Rahmann, S. (2012). Snakemake--a scalable bioinformatics workflow engine. Bioinformatics 28, 2520-2522.
14. Li, H., and Durbin, R. (2009). Fast and accurate short read alignment with Burrows-Wheeler transform. Bioinformatics 25, 1754-1760.
15. Dobin, A., Davis, C.A., Schlesinger, F., Drenkow, J., Zaleski, C., Jha, S., Batut, P., Chaisson, M., and Gingeras, T.R. (2013). STAR: ultrafast universal RNA-seq aligner. Bioinformatics 29, 15-21.

16. Quinlan, A.R., and Hall, I.M. (2010). BEDTools: a flexible suite of utilities for comparing genomic features. Bioinformatics 26, 841-842.
17. Rimmer, A., Phan, H., Mathieson, I., Iqbal, Z., Twigg, S.R.F., Consortium, W.G.S., Wilkie, A.O.M., McVean, G., and Lunter, G. (2014). Integrating mapping-, assembly- and haplotype-based approaches for calling variants in clinical sequencing applications. Nat Genet 46, 912-918.
18. Wang, K., Li, M., and Hakonarson, H. (2010). ANNOVAR: functional annotation of genetic variants from high-throughput sequencing data. Nucleic Acids Res 38, e164.
19. Heger A, B.T., Finkernagel F, Goodstadt L, Goodson M, Jacobs KB, Lunter G, Martin M, Schiller B. Pysam: Python Interface for the SAM/BAM Sequence Alignment and Mapping Format. https://githubcom/pysam-developers/pysam Accessed 18-08-2017.
20. Cock, P.J., Antao, T., Chang, J.T., Chapman, B.A., Cox, C.J., Dalke, A., Friedberg, I., Hamelryck, T., Kauff, F., Wilczynski, B., et al. (2009). Biopython: freely available Python tools for computational molecular biology and bioinformatics. Bioinformatics 25, 1422-1423.
21. McKinney, W. (2010). Data structures for statistical computing in Python. in Proceedings of the 9th Python in Science Conference, 51-56.
22. Ferry, Q., Steinberg, J., Webber, C., FitzPatrick, D.R., Ponting, C.P., Zisserman, A., and Nellaker, C. (2014). Diagnostically relevant facial gestalt information from ordinary photos. eLife 3, e02020.
23. Ansari, M., Poke, G., Ferry, Q., Williamson, K., Aldridge, R., Meynert, A.M., Bengani, H., Chan, C.Y., Kayserili, H., Avci, S., et al. (2014). Genetic heterogeneity in Cornelia de Lange syndrome (CdLS) and CdLS-like phenotypes with observed and predicted levels of mosaicism. J Med Genet 51, 659-668.
24. Kazemi, V., Sullivan, J. (2014). One millisecond face alignment with an ensemble of regression trees. Proceedings of the IEEE Computer Society Conference on Computer Vision and Pattern Recognition, 1867–1874.
