# Supplementary material for: De Novo and Inherited Loss-of-Function Variants in TLK2: Clinical and Genotype-Phenotype Evaluation of a Distinct Neurodevelopmental Disorder
Source: Am J Hum Genet. 2018 May 31;102(6):1195–203. doi: 10.1016/j.ajhg.2018.04.014 (PMC5992133; doi:10.1016/j.ajhg.2018.04.014)
Supplement: Document S2. Article plus Supplemental Data [file mmc2.pdf]

Please cite this article in press as: Reijnders et al., *De Novo* and Inherited Loss-of-Function Variants in *TLK2*: Clinical and Genotype-Phenotype Evaluation of a Distinct Neurod..., The American Journal of Human Genetics (2018), https://doi.org/10.1016/j.ajhg.2018.04.014

REPORT

De Novo and Inherited Loss-of-Function Variants in *TLK2*: Clinical and Genotype-Phenotype Evaluation of a Distinct Neurodevelopmental Disorder

Margot R.F. Reijnders,[1,54] Kerry A. Miller,[2,54] Mohsan Alvi,[3] Jacqueline A.C. Goos,[4] Melissa M. Lees,[5] Anna de Burca,[6] Alex Henderson,[7] Alison Kraus,[8] Barbara Mikat,[9] Bert B.A. de Vries,[1] Bertrand Isidor,[10,11] Bronwyn Kerr,[12,13] Carlo Marcelis,[14] Caroline Schluth-Bolard,[15,16] Charu Deshpande,[17] Claudia A.L. Ruivenkamp,[18] Dagmar Wieczorek,[9,19] The Deciphering Developmental Disorders Study,[20] Diana Baralle,[21,22] Edward M. Blair,[6] Hartmut Engels,[23] Hermann-Josef Lüdecke,[9,19] Jacqueline Eason,[24] Gijs W.E. Santen,[18] Jill Clayton-Smith,[12,13] Kate Chandler,[12,13] Katrina Tatton-Brown,[25] Katelyn Payne,[26] Katherine Helbig,[27] Kelly Radtke,[27]

(Author list continued on next page)

Next-generation sequencing is a powerful tool for the discovery of genes related to neurodevelopmental disorders (NDDs). Here, we report the identification of a distinct syndrome due to *de novo* or inherited heterozygous mutations in Tousled-like kinase 2 (*TLK2*) in 38 unrelated individuals and two affected mothers, using whole-exome and whole-genome sequencing technologies, matchmaker databases, and international collaborations. Affected individuals had a consistent phenotype, characterized by mild-borderline neurodevelopmental delay (86%), behavioral disorders (68%), severe gastro-intestinal problems (63%), and facial dysmorphism including blepharophimosis (82%), telecanthus (74%), prominent nasal bridge (68%), broad nasal tip (66%), thin vermilion of the upper lip (62%), and upslanting palpebral fissures (55%). Analysis of cell lines from three affected individuals showed that mutations act through a loss-of-function mechanism in at least two case subjects. Genotype-phenotype analysis and comparison of computationally modeled faces showed that phenotypes of these and other individuals with loss-of-function variants significantly overlapped with phenotypes of individuals with other variant types (missense and C-terminal truncating). This suggests that haploinsufficiency of TLK2 is the most likely underlying disease mechanism, leading to a consistent neurodevelopmental phenotype. This work illustrates the power of international data sharing, by the identification of 40 individuals from 26 different centers in 7 different countries, allowing the identification, clinical delineation, and genotype-phenotype evaluation of a distinct NDD caused by mutations in *TLK2*.

The introduction of whole-exome sequencing (WES) as a diagnostic test for individuals with unexplained neurodevelopmental disorders (NDDs) has led to the identification of dozens of disease-associated genes. As a recent example, statistical analysis of aggregated exome data uncovered variants in ten different genes as likely causes of intellectual disability, a subtype of NDDs characterized by deficits in both intellectual and adaptive functioning.[1,2] One such gene was Tousled-like kinase 2 (*TLK2* [MIM: 608439]), which was originally named because of homology to the

[1]Department of Human Genetics, Donders Institute for Brain, Cognition, and Behaviour, Radboud University Medical Center, Nijmegen, 6500 HB, the Netherlands; [2]Clinical Genetics Group, MRC Weatherall Institute of Molecular Medicine, University of Oxford, John Radcliffe Hospital, Oxford OX3 9DS, UK; [3]Visual Geometry Group, Department of Engineering Science, University of Oxford, Oxford OX1 2JD, UK; [4]Department of Plastic and Reconstructive Surgery, Erasmus MC, University Medical Center Rotterdam, PO Box 2040, 3000 CA Rotterdam, the Netherlands; [5]Department of Clinical Genetics, Great Ormond Street Hospital, London WC1N 3JH, UK; [6]Oxford Centre for Genomic Medicine, Oxford University Hospitals NHS Foundation Trust, Oxford OX3 7HE, UK; [7]Northern Genetics Service, Newcastle upon Tyne Hospitals NHS Foundation Trust, Newcastle upon Tyne NE1 3BZ, UK; [8]Yorkshire Regional Genetics Service, Chapel Allerton Hospital, Leeds LS7 4SA, UK; [9]Institut für Humangenetik, Universitätsklinikum Essen, Universität Duisburg-Essen, 45147 Essen, Germany; [10]CHU de Nantes, Service de Génétique Médicale, Nantes 44093 Cedex 1, France; [11]INSERM, UMR-S 957, 1 Rue Gaston Veil, Nantes 44035, France; [12]Division of Evolution and Genomic Sciences, School of Biological Sciences, University of Manchester, Manchester M13 9PL, UK; [13]Manchester Centre for Genomic Medicine, Manchester University Hospitals NHS Foundation Trust, Manchester Academic Health Sciences Centre, Manchester M13 9WL, UK; [14]Department of Human Genetics, Radboud University Medical Center, Nijmegen 6500 HB, the Netherlands; [15]Hospices Civils de Lyon, Service de Génétique, Centre de Référence Anomalies du Développement, 69500 Bron, France; [16]INSERM U1028, CNRS UMR5292, UCB Lyon 1, Centre de Recherche en Neurosciences de Lyon, GENDEV Team, 69500 Bron, France; [17]South East Thames Regional Genetics Service, Guy's Hospital, London SE1 9RT, UK; [18]Department of Clinical Genetics, Leiden University Medical Center, 2300 RC Leiden, the Netherlands; [19]Institute of Human Genetics, Heinrich-Heine-University, Medical Faculty, 40225 Düsseldorf, Germany; [20]Wellcome Trust Sanger Institute, Hinxton CB10 1SA, UK; [21]Human Development and Health, Duthie Building, University of Southampton, Southampton SO16 6YD, UK; [22]Wessex Clinical Genetics Service, Princess Anne Hospital, Southampton SO16 5YA, UK; [23]Institute of Human Genetics, University of Bonn, School of Medicine & University Hospital Bonn, 53127 Bonn, Germany; [24]Nottingham Regional Genetics Service, City Hospital Campus, Nottingham University Hospitals NHS Trust, Hucknall Road, Nottingham NG5 1PB, UK; [25]Southwest Thames Regional Genetics Centre, St George's University Hospitals NHS Foundation Trust, St George's University of London, London SW17 0RE, UK; [26]Riley Hospital for Children, Indianapolis, Indiana, IN 46202, USA; [27]Division of Clinical Genomics, Ambry Genetics, Aliso Viejo, CA 92656, USA; [28]Department of Pediatrics, Baylor College of Medicine, The Children's Hospital of San Antonio, San Antonio, TX 78207, USA; [29]Department of Molecular and Human Genetics, Baylor College of Medicine, Houston, TX 77030, USA; [30]Institute of Human Genetics, Helmholtz Zentrum München, 85764 Neuherberg, Germany; [31]Institute of Human Genetics, Technische Universität München, 81675 Munich, Germany; [32]University of California, San Diego,

(Affiliations continued on next page)

© 2018 The Authors. This is an open access article under the CC BY license (http://creativecommons.org/licenses/by/4.0/).

Kimberly M. Nugent,[28,29] Kirsten Cremer,[23] Tim M. Strom,[30,31] Lynne M. Bird,[32] Margje Sinnema,[33] Maria Bitner-Glindzicz,[34] Marieke F. van Dooren,[35] Marielle Alders,[36] Marije Koopmans,[18,37] Lauren Brick,[38] Mariya Kozenko,[38] Megan L. Harline,[28] Merel Klaassens,[39] Michelle Steinraths,[40] Nicola S. Cooper,[41] Patrick Edery,[15,16] Patrick Yap,[42,43,44] Paulien A. Terhal,[37] Peter J. van der Spek,[45] Phillis Lakeman,[36] Rachel L. Taylor,[12,13] Rebecca O. Littlejohn,[28,29] Rolph Pfundt,[1] Saadet Mercimek-Andrews,[46] Alexander P.A. Stegmann,[33] Sarina G. Kant,[18] Scott McLean,[28,29] Shelagh Joss,[47] Sigrid M.A. Swagemakers,[45] Sofia Douzgou,[12,13] Steven A. Wall,[48] Sébastien Küry,[49] Eduardo Calpena,[2] Nils Koelling,[2] Simon J. McGowan,[50] Stephen R.F. Twigg,[2] Irene M.J. Mathijssen,[4] Christoffer Nellaker,[51,52,53] Han G. Brunner,[1,33,54,*] and Andrew O.M. Wilkie[2,48,54,*]

Arabidopsis gene *Tousled*.[3] *TLK2*, ubiquitously expressed in all tissues including fetal brain, encodes a serine/threonine kinase comprising a catalytic domain and multiple highly conserved coiled-coil motifs.[3,4] TLK2 is known to have maximal activity during the S-phase of the cell cycle and is therefore tightly linked to DNA replication.[3] DNA double-strand breaks lead to rapid and transient inhibition of TLK activity, suggesting a role in checkpoint regulation.[5] With the discovery of both H3-H4 chaperone Asf1 and histone H3 as physiological substrates of TLKs, its protein function has been linked to chromatin assembly.[6–10]

To establish the contribution of *TLK2* variants to NDDs in humans, we systematically collected phenotypic data of the five affected individuals with *TLK2* variants reported previously,[1] derived cell lines, and exploited different strategies to identify additional individuals with a variant in this gene. By including *TLK2* in a Deciphering Developmental Disorders[11] Complementary Analysis Project, by using of GeneMatcher,[12] and by sharing data with international collaborators, we identified a total of 38 unrelated individuals and two affected mothers with heterozygous variants in *TLK2*. Variants were detected by either family-based WES (research settings, n = 18 probands and 2 affected parents; diagnostic settings, n = 18 probands) or whole-genome sequencing (WGS) (research settings, n = 2 probands) in 26 different institutions and 7 different countries (Figure S1; Supplemental Subjects and Methods). Two additional individuals with *de novo TLK2* variants c.1514T>A (p.Val505Asp) and c.2171G>A (p.Arg724Gln), each of whom had a second likely pathogenic mutation in another gene, were excluded from further consideration to avoid confounding in the phenotypic analysis (Supplemental Subjects and Methods). IRB-approved consents for WES or WGS in diagnostic or research settings were obtained for all individuals.

We observed a broad spectrum of different variant types in *TLK2* (GenBank: NM_006852): 4 frameshift variants, 10 nonsense variants (including 2 located in the last exon), 12 canonical splice-site variants, and 9 missense variants (Figures 1A–1C; Table 1). Additionally, we identified a *de novo* balanced translocation in one of the WGS case subjects, resulting in a breakpoint at chromosome 17q23.2 disrupting the *TLK2* intron between exons 2 and 3 (Figure 1D; Supplemental Subjects and Methods). Interestingly, we found recurrent mutations within our cohort of affected individuals, occurring at hypermutable sites as reported by Rahbari et al.[13] We considered the alternative possibility of gene conversion, because pseudogenes very similar to *TLK2* exist at 10p11.21 and/or 17q12; however, the pseudogene sequence(s) at the site of each recurrent mutation correspond to wild-type *TLK2*, excluding this mechanism. The missense variants c.1487A>G (p.His496Arg) and c.1015C>T (p.Arg339Trp) were each identified in two unrelated individuals, and c.1016G>A (p.Arg339Gln) also occurs at the Arg339 codon (Figure 1C; Table 1). Finally, two splice variants were predicted to give rise to the same

Department of Pediatrics; Genetics and Dysmorphology, Rady Children's Hospital San Diego, San Diego, CA 92123, USA; [33]Department of Clinical Genetics and School for Oncology & Developmental Biology (GROW), Maastricht University Medical Center, Maastricht 6229 ER, the Netherlands; [34]Genetics and Genomic Medicine, UCL Great Ormond Street Institute of Child Health, 30 Guilford Street, London WC1N 1EH, UK; [35]Department of Clinical Genetics, Erasmus MC, University Medical Center Rotterdam, PO Box 21455, 3001 AL Rotterdam, the Netherlands; [36]Department of Clinical Genetics, Academic Medical Center, PO Box 22660, 1100 DD Amsterdam, the Netherlands; [37]Department of Genetics, University Medical Center Utrecht, 3508 AB Utrecht, the Netherlands; [38]Division of Genetics, Department of Pediatrics, McMaster Children's Hospital, McMaster University, Hamilton, ON L8N 3Z5, Canada; [39]Department of Paediatrics, Maastricht University Medical Center, Maastricht 6229 ER, the Netherlands; [40]Department of Medical Genetics, University of British Columbia, Vancouver, BC V8Z 6R5, Canada; [41]West Midlands Regional Clinical Genetics Unit, Birmingham Women's & Children's NHS Foundation Trust, Mindelsohn Way, Birmingham B15 2TG, UK; [42]Genetic Health Service New Zealand, Auckland 1142, New Zealand; [43]Victorian Clinical Genetic Services, Murdoch Children's Research Institute, Melbourne, VIC 3052, Australia; [44]University of Auckland, Auckland 1142, New Zealand; [45]Department of Pathology & Department of Bioinformatics, Erasmus MC, University Medical Center Rotterdam, PO Box 2040, 3000 CA Rotterdam, the Netherlands; [46]Division of Clinical and Metabolic Genetics, Department of Pediatrics, University of Toronto, Toronto, ON, Canada; Genetics and Genome Biology Program, Research Institute, The Hospital for Sick Children, Toronto, ON, Canada; Institute of Medical Sciences, University of Toronto, Toronto, ON M5G 1X8, Canada; [47]West of Scotland Clinical Genetics Service, Queen Elizabeth University Hospital, Glasgow G51 4TF, UK; [48]Craniofacial Unit, Oxford University Hospitals NHS Trust, John Radcliffe Hospital, Oxford OX3 9DU, UK; [49]CHU de Nantes, Service de Génétique Médicale, 44093 Nantes Cedex 1, France; [50]Computational Biology Research Group, MRC Weatherall Institute of Molecular Medicine, University of Oxford, John Radcliffe Hospital, Oxford OX3 9DS, UK; [51]Nuffield Department of Women's and Reproductive Health, University of Oxford, Women's Centre, John Radcliffe Hospital, Oxford OX3 9DS, UK; [52]Institute of Biomedical Engineering, Department of Engineering Science, University of Oxford, Oxford OX3 7FZ, UK; [53]Big Data Institute, Li Ka Shing Centre for Health Information and Discovery, University of Oxford, Oxford OX3 7FZ, UK
[54]These authors contributed equally to this work
*Correspondence: han.brunner@radboudumc.nl (H.G.B.), andrew.wilkie@imm.ox.ac.uk (A.O.M.W.)
https://doi.org/10.1016/j.ajhg.2018.04.014.

Please cite this article in press as: Reijnders et al., *De Novo* and Inherited Loss-of-Function Variants in *TLK2*: Clinical and Genotype-Phenotype Evaluation of a Distinct Neurod..., The American Journal of Human Genetics (2018), https://doi.org/10.1016/j.ajhg.2018.04.014

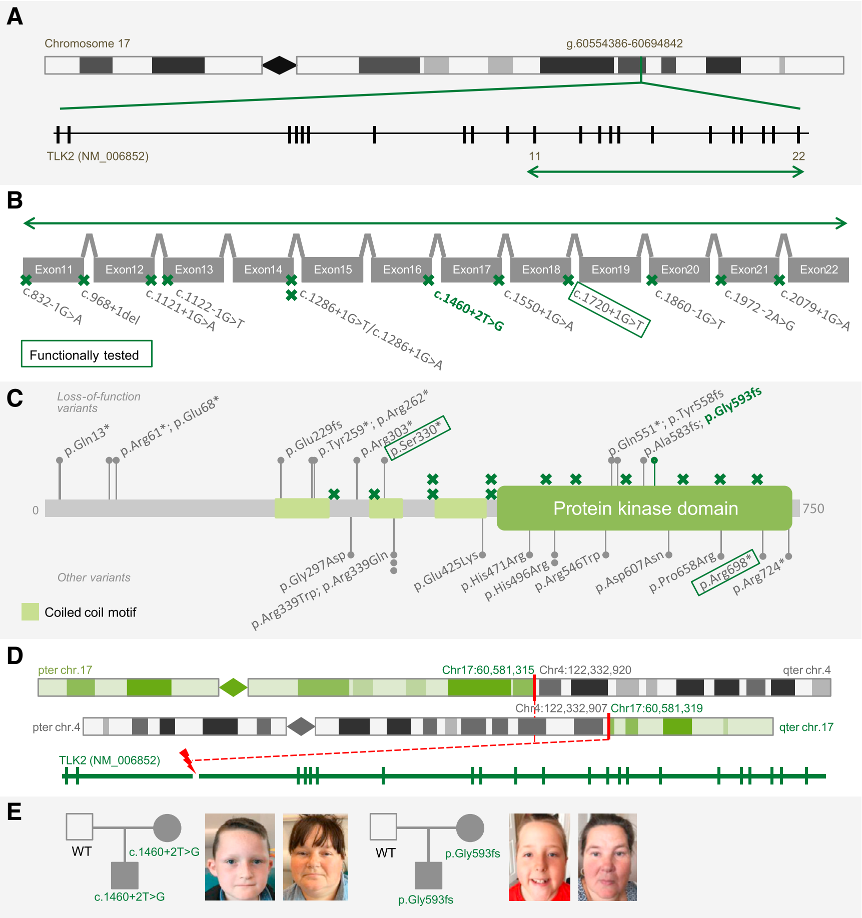

Figure 1. Intragenic Variants and Balanced Translocation Identified in *TLK2*

(A) Location of *TLK2* (GenBank: NM_006852.3) on chromosome 17q23.2 (see Supplemental Subjects and Methods for discussion about different *TLK2* spliceforms). Vertical marks in *TLK2* represent the 22 exons. Green arrow indicates region enlarged in panel below.

(B) Schematic view (not to scale) of exons 11–22 and locations of 12 identified splice site mutations (green crosses). The splice site mutation inherited from an affected parent is shown in bold and green. The variant subjected to cDNA analysis is shown in the dark green rectangle.

(C) Overview of TLK2 protein with the protein kinase domain (dark green) and three coiled-coil motifs (light green). Loss-of-function variants (24 total, including 8 nonsense, 4 frameshift, and 12 splice site mutations) are shown above the protein with green crosses indicating positions of splice site mutations. Other variants (11 missense variants and 2 nonsense variants causing a premature stop codon in the last exon) are shown below the protein. The frameshift mutation inherited from an affected parent is shown in bold and green. The variants subjected to cDNA analysis are shown in the dark green rectangles.

(D) Balanced translocation between chromosomes 4 and 17, with the breakpoint disrupting *TLK2* between exons 2 and 3, identified in one individual: 46,XX,t(4;17)(27;q23.2).seq[GRCh37]t(4;17)g.[chr4:pter_cen_122332907:: chr17:60,581,319_qter]g.[chr17_pter_cen_60,581,315::chr4:122,332,920_qter].

(E) Pedigrees of individuals with inherited variants and photographs of probands and their affected mothers. Both mothers have facial dysmorphism similar to their children. WT, wild-type at variant position.

affected protein product: c.1286+1G>T and c.1286+1G>A (Figure 1B; Table 1). From the 9 missense variants identified in 11 unrelated individuals, 5 are located in the catalytic domain of the protein and 3 in a coiled-coil motif. One variant, c.890G>A (p.Gly297Asp), is located outside a known functional domain, but affects a highly conserved amino acid and was predicted pathogenic by several *in silico* prediction programs, similar to other missense variants (Figure 1C; Table S1). None of the missense variants were present in the ExAC database,[14] nor in our in-house database of variants identified in healthy control subjects. The recently released gnomAD database, containing WGS variants identified in control subjects, reported only c.1636C>T (p.Arg546Trp) in a single individual (allele frequency of ~0.000004). None of the other missense variants were present in the gnomAD database (Table 1).

For all but two variants (Table 1), the *de novo* status was assessed by sequencing the parents of the proband. In two individuals, variants were inherited from a similarly affected parent, while all other variants (n = 34) occurred *de novo*. Detailed phenotyping revealed that both mothers carrying a predicted loss-of-function (LOF) *TLK2* variant (Table 1) were mildly affected. The first mother (c.1460+2T>G) had mild neurodevelopmental delay and speech delay. The second affected mother (c.1776_1783delTGGTCTTT [p.Gly593Glufs*5]) had a low-normal IQ level but was diagnosed with bipolar disorder. Both had facial dysmorphism similar to their affected children (Figure 1E). The inherited variants illustrate that the search for a diagnosis should not always be restricted to *de novo* mutations, in particular if individuals are only mildly affected. Similar to the parents in this study, who were never referred for genetic testing before investigation of their child uncovered a *TLK2* variant, we expect mutations causing milder phenotypes to be present in the general population. This could explain why, although *TLK2* exhibits very strong constraint against LOF variants (pLI = 1), five LOF variants (low-coverage variants excluded) have been reported in gnomAD, and a missense variant—c.1636C>T (p.Arg546Trp)—that was reported here as *de novo* variant, was present at very low allele frequency in the population (aggregate minor allele frequency of LOF and missense variants ~0.000024).

Table 1. Intragenic Variants in *TLK2* (GenBank: NM_006852.3), Inheritance, and Presence in ExAC and gnomAD Databases

Subgroup	cDNA Position	Protein Position	Inheritance	RNA Analysis	cMAF ExAC	cMAF gnomAD
Predicted LOF	c.37C>T	p.Gln13*	*de novo*	no	no LOF variants	5 LOF variants: ∼0.00002
	c.181C>T	p.Arg61*	*de novo*	no		
	c.202G>T	p.Glu68*	*de novo*	no		
	c.685_688del	p.Glu229Argfs*6	*de novo*	no		
	c.777C>A	p.Tyr259*	*de novo*	no		
	c.784C>T	p.Arg262*	*de novo*	no		
	c.832−1G>A	unknown	*de novo*	no		
	c.907C>T	p.Arg303*	*de novo*	no		
	c.968+1del	unknown	*de novo*	no		
	c.989C>A	p.Ser330*	*de novo*	yes		
	c.1121+1G>A	unknown	*de novo*	no		
	c.1122−1G>T	unknown	*de novo*	no		
	c.1286+1G>T	unknown	*de novo*	no		
	c.1286+1G>A	unknown	*de novo*	no		
	c.1460+2T>G	unknown	inherited	no		
	c.1550+1G>A	unknown	*de novo*	no		
	c.1651C>T	p.Gln551*	*de novo*	no		
	c.1672dup	p.Tyr558Leufs*4	*de novo*	no		
	c.1720+1G>T[a]	unknown	*de novo*	yes		
	c.1746delA	p.Ala583Argfs*5	*de novo*	no		
	c.1776_1783delTGGTCTTT	p.Gly593Glufs*5	inherited	no		
	c.1860−1G>T	unknown	unknown	no		
	c.1972−2A>G	unknown	*de novo*	no		
	c.2079+1G>A	unknown	*de novo*	no		
Other variant types	c.2092C>T[a]	p.Arg698*	*de novo*	yes	0	0
	c.2170C>T	p.Arg724*	*de novo*	no	0	0
	c.890G>A	p.Gly297Asp	*de novo*	no	0	0
	c.1015C>T	p.Arg339Trp	*de novo*[b]	no	0	0
	c.1016G>A	p.Arg339Gln	*de novo*	no	0	0
	c.1273G>A	p.Glu425Lys	unknown	no	0	0
	c.1412A>G[a]	p.His471Arg	*de novo*	no	0	0
	c.1487A>G[a]	p.His496Arg	*de novo*[b]	no	0	0
	c.1636C>T	p.Arg546Trp	*de novo*	no	0	∼0.000004
	c.1819G>A[a]	p.Asp607Asn	*de novo*	no	0	0
	c.1973C>G	p.Pro658Arg	*de novo*	no	0	0

Identified balanced translocation (n = 1) is not included in this table. Abbreviations: cMAF, cumulative minor allele frequency; LOF, loss-of-function
[a]Variant reported previously[1]
[b]Recurrent *de novo* variant identified in two unrelated individuals

Consistent with the phenotypes of both affected mothers, mild neurodevelopmental phenotypes accompanied by language and motor delay were present in the majority of the 38 unrelated probands: 6% of the individuals had normal IQ levels (85–100), 14% had borderline ID (IQ 70–85), and from the 72% diagnosed with ID (IQ < 70), most had mild ID (IQ 50–70) (Figure 2). Most of the affected probands (22 males and 16 females) were children at the time of last examination (median 8.0 years; interquartile range 4.1–13.5 years); ages ranged between

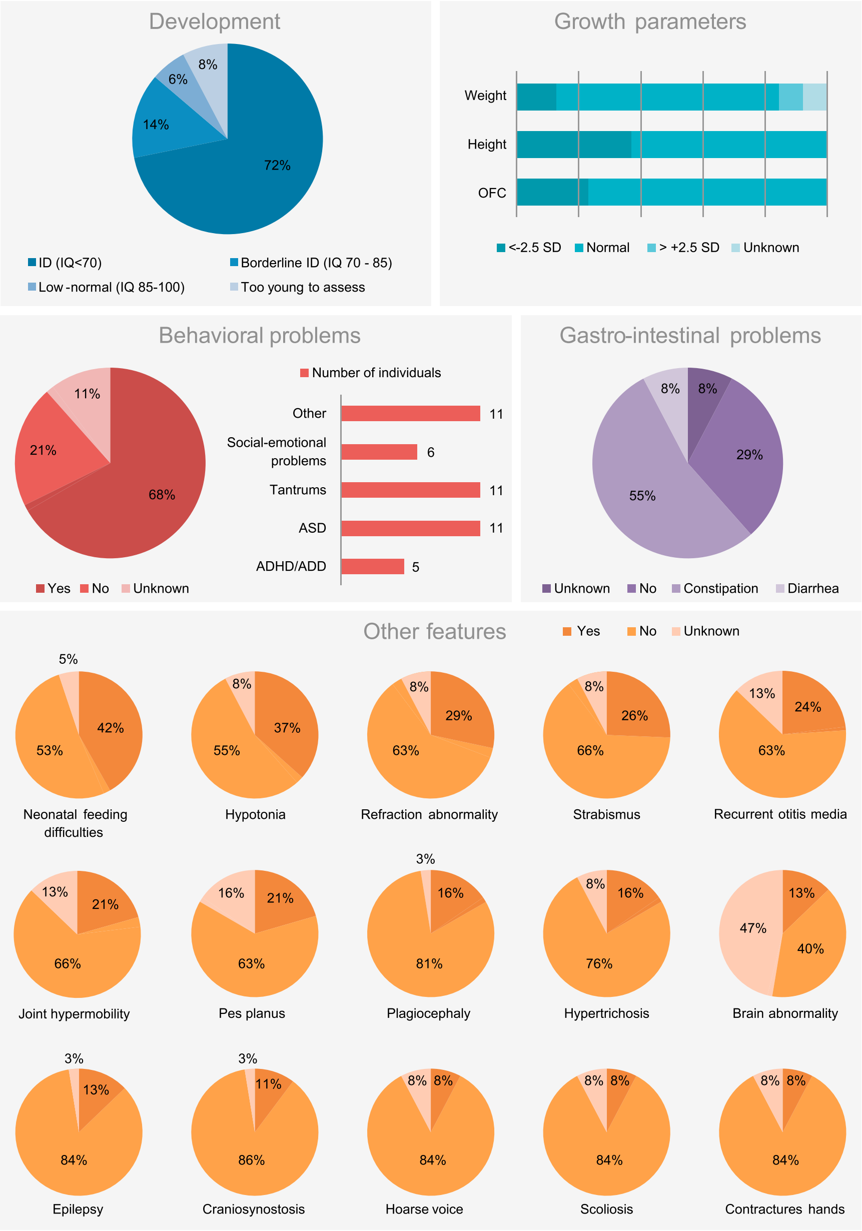

Figure 2. Clinical Spectrum Associated with *TLK2* Variants
Overview of clinical features observed in individuals with *TLK2* variants.

3 months and 29 years. Three individuals, who all had language and motor delay, were too young for formal assessment of their neurodevelopmental phenotype. In addition to this, systematic evaluation of other clinical data, scored by the referring clinician, showed a variety of overlapping features (Figure 2, Table S2). Neurological problems including hypotonia (37%), epilepsy (13%), and non-specific intracranial brain abnormalities (13%) (Table S3) were observed. A broad range of behavioral disorders was present (68%), with often severely affected social functioning: tantrums (11 individuals), autism spectrum disorder (ASD; 11 individuals), attention-deficit disorder with or without hyperactivity (ADHD; 5 individuals), and severe social-emotional problems (6 individuals) were the most commonly reported problems. Less frequently observed were short attention span, pica disorder, aggression, obsessive-compulsive disorder, and anxiety in 11 individuals. Other recurrent features included gastro-intestinal problems (constipation in 55%; severe diarrhea in 8%), neonatal feeding difficulties (42%), eye abnormalities (refraction abnormality in 29%, strabismus in 26%), musculoskeletal abnormalities (joint hypermobility in 21%; pes planus in 21%; toe walking in 18%; scoliosis in 8%; contractures of the hands in 8%), recurrent otitis media (24%), hypertrichosis (16%), and hoarse voice (8%). Abnormalities of skull shape were observed in 31% of probands (Figure 2, Tables S2 and S4), with clinically proven craniosynostosis being present in four (11%) of them (Table S5). However, sequence-based screening of 309 DNA samples from individuals with mixed, genetically undiagnosed craniosynostosis (Supplemental Subjects and Methods, Table S6) did not identify further case subjects, indicating that *TLK2* mutations are a rare cause of craniosynostosis. Growth parameters were frequently abnormal (Figure 2). Short stature was documented in 37%, microcephaly in 24% (primary in 13%, secondary in 3%, and unknown age of onset in 8%), and low body weight in 13%. Three individuals (8%) were overweight, with age of onset between the ages of 2 and 12 years. Features reported in only one or two individuals are summarized in Table S4. In addition to the other clinical features, overlapping facial dysmorphisms were present (Figures 3A and 3B). Most frequently reported by clinicians were blepharophimosis (82%), telecanthus (74%), prominent nasal bridge (68%), broad nasal tip (66%), thin vermilion of the upper lip (62%), and upslanting palpebral fissures (55%). Pointed and tall chin (42%), epicanthal folds (42%), narrow mouth (32%), high palate (30%), microtia, first degree (29%), posteriorly rotated ears (29%), long face (27%), ptosis (21%), and asymmetric face (16%) were observed in fewer than half of the individuals.

Analysis of data from the ExAC database demonstrates that *TLK2* is extremely intolerant for LOF variants (pLI score = 1).[14] In line with this observation, animal models

Please cite this article in press as: Reijnders et al., *De Novo* and Inherited Loss-of-Function Variants in *TLK2*: Clinical and Genotype-Phenotype Evaluation of a Distinct Neurod..., The American Journal of Human Genetics (2018), https://doi.org/10.1016/j.ajhg.2018.04.014

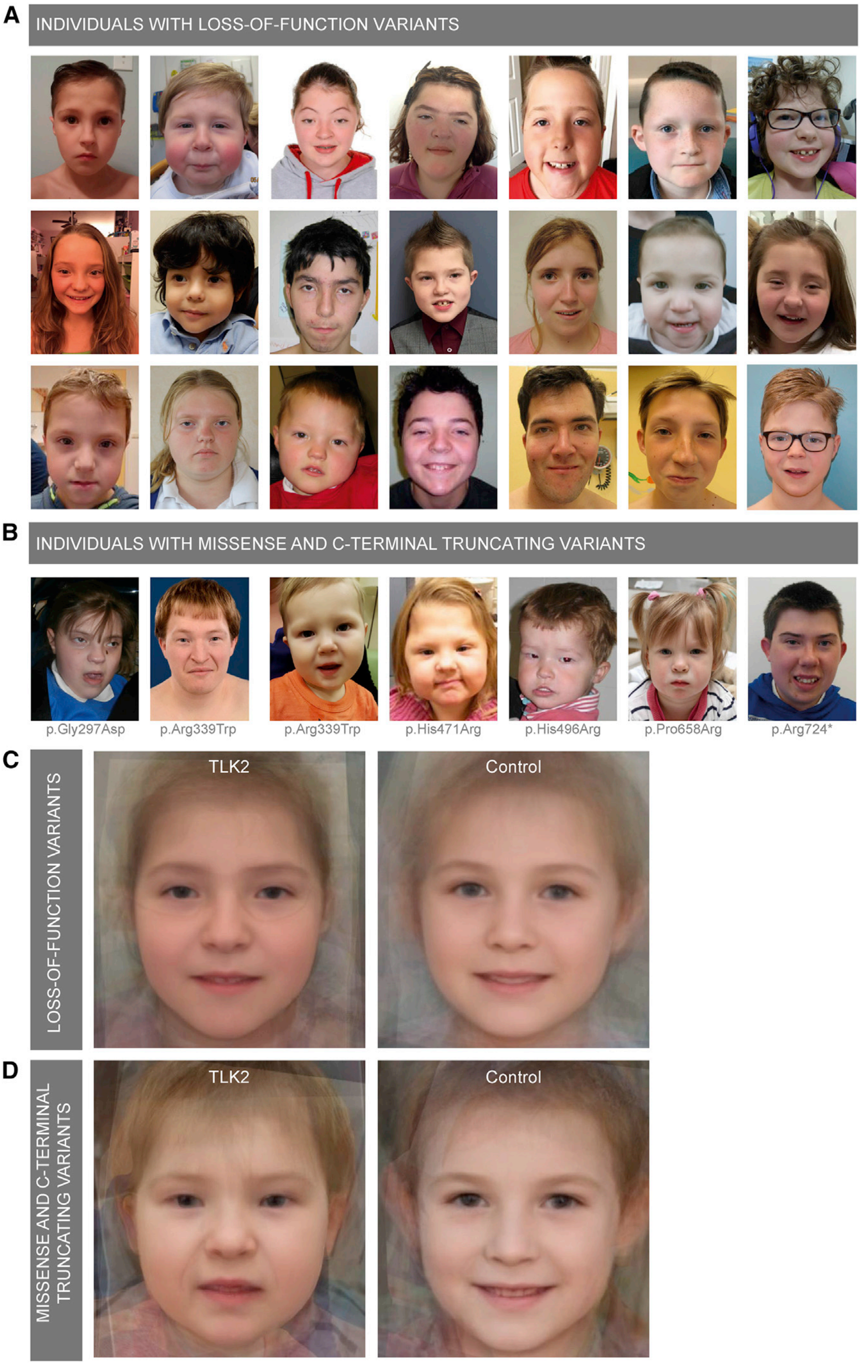

Figure 3. Facial Dysmorphism of Individuals with *TLK2* Variants

(A) Photographs of 21 unrelated individuals with a loss-of-function variant in *TLK2*, showing overlapping facial dysmorphism. Most frequently reported by clinicians were blepharophimosis, telecanthus, prominent nasal bridge, broad nasal tip, thin vermilion upper lip, and upward slanted palpebral fissures. Pointed and tall chin, epicanthal folds, narrow mouth, high palate, microtia, posteriorly rotated ears, long face, ptosis, and asymmetric face were observed in fewer than half of the individuals.

(B) Photographs of seven unrelated individuals with a missense or C-terminal truncating variant in TLK2. Variant c.2170C>T (p.Arg724*) is assigned to this subgroup, since a premature stop codon is introduced in the last exon. Facial dysmorphisms overlapped with dysmorphism observed in individuals with loss-of-function variants.

(C) Computational averaging of 33 facial photographs of 22 subjects with LOF variants in *TLK2* (left) compared with 22 gender- and age-matched control subjects (right).

(D) Computational averaging of 11 facial photographs of 8 subjects with missense or C-terminal truncating variants in *TLK2* (left) compared with 8 gender- and age-matched control subjects (right).

with depletion of TLK2 have been reported to have severely disturbed cellular and developmental processes. *Drosophila* with complete LOF of TLK were associated with arrested nuclear divisions, causing apoptosis of the cell.[7] *Tlk2*-null mice were embryonically lethal due to placental failure.[15] In this study, we found several predicted LOF variants in affected individuals. To investigate whether variants resulted in an aberrant transcript, we synthesized cDNA from RNA extracted from fibroblast or lymphoblastoid cell lines (Supplemental Subjects and Methods, Table S7) from three individuals with different variants: (1) c.989C>A (p.Ser330*), predicted to result in a truncated product leading to nonsense-mediated decay (NMD); (2) c.2092C>T (p.Arg698*), with a premature stop codon in the last exon predicted to escape from NMD; and (3) c.1720+1G>T, a mutation predicted to affect splicing of exon 18. To investigate the significance of NMD for expression of *TLK2* transcripts, we treated fibroblasts (for p.Ser330*) and lymphoblastoid cell lines (for p.Ser330*, p.Arg698*, and c.1720+1G>T) with cycloheximide, an inhibitor of NMD.[16] Transcript stability of cDNA PCR products from p.Ser330* and p.Arg698* individuals in the presence of cycloheximide was analyzed using a restriction enzyme assay targeting the wild-type transcript and the results were confirmed using deep sequencing to quantify relative levels of wild-type and mutant transcripts (Supplemental Subjects and Methods). For fibroblast and lymphoblastoid cell lines heterozygous for the p.Ser330* variant, the mutant allele represented 15.8% and 21.5% of transcripts, respectively, in the absence of cycloheximide, but rose to 37.7% and 48.5%, respectively, in the presence of cycloheximide, supporting that this variant is subject to NMD and causes haploinsufficiency of TLK2. In contrast, wild-type and mutant transcripts

Please cite this article in press as: Reijnders et al., *De Novo* and Inherited Loss-of-Function Variants in *TLK2*: Clinical and Genotype-Phenotype Evaluation of a Distinct Neurod..., The American Journal of Human Genetics (2018), https://doi.org/10.1016/j.ajhg.2018.04.014

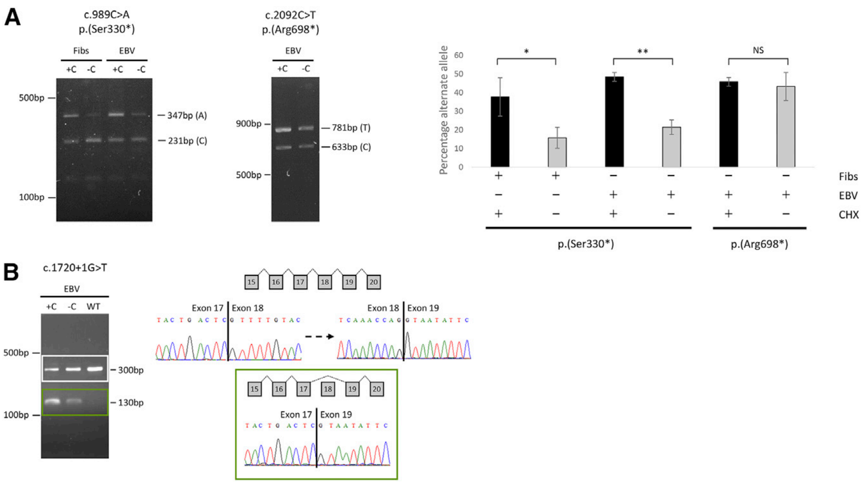

Figure 4. Analysis of *TLK2* Transcripts in Cell Lines

(A) Analysis of transcripts encoding nonsense mutations c.989C>A (p.Ser330*) and c.2092C>T (p.Arg698*) in cell lines of affected individuals. Left panel shows reverse transcriptase-PCR (RT-PCR) products of cDNA prepared from fibroblast and lymphoblastoid cell lines of subject with p.Ser330* variant, either in the presence (+C) or absence (−C) of cycloheximide and incubated with ApoI (digests wild-type allele). Central panel shows RT-PCR of cDNA prepared from lymphoblastoid cell line of subject with p.Arg698* variant, in the presence (+C) or absence (−C) of cycloheximide and incubated with Hpy99I (digests wild-type allele). Right panel shows proportion (± standard deviation) of variant alleles quantified by deep sequencing of triplicate samples. Statistical testing of differences: $^{*}p = 0.046$; $^{**}p = 0.011$; NS, not significant.

(B) Analysis of transcripts with canonical splice-site mutation c.1720+1G>T. A wild-type fragment at 300 bp in c.1720+1G>T lymphoblastoid cells is observed as well as a second fragment at 130 bp, which is absent in control cDNA. An increase of mutant transcript in cells was present when treated with cycloheximide (+C), indicating that the aberrant transcript was subject to NMD. Sequencing of the 300 bp (white box) and 130 bp (green box) fragments demonstrated skipping of exon 18 in the lower cDNA product. Abbreviations: Fibs, fibroblasts; EBV, lymphoblastoid cells; C/CHX, cycloheximide; WT, control cDNA.

from lymphoblastoid cells of the individual heterozygous for p.Arg698* did not show significant differences between treated and untreated cells, supporting that the mutant transcript escapes NMD due to its location within the last coding exon of *TLK2* (Figure 4A). Amplification of cDNA from an individual with a splice-site variant (c.1720+1G>T) showed a full-length wild-type product of 300 bp and an additional aberrant smaller product of 130 bp, consistent with skipping of exon 18. Direct sequencing of this smaller fragment confirmed that exon 17 spliced directly to exon 19, thereby producing an out-of-frame transcript predicted to introduce a premature stop codon at the next amino acid position (p.Ser517fs*1). Additionally, the intensity of the spliced transcript increased when treated with cycloheximide, indicating that the mutant transcript is subjected to NMD (Figure 4B).

By analyzing *TLK2* transcripts in cell lines of three different individuals, we were able to confirm that transcripts were subjected to NMD in two of them, causing haploinsufficiency of TLK2. It is likely that comparable variants predicted to cause LOF of TLK2 affect the transcript similarly. The large number of identified individuals with *TLK2* variants allowed us to search for underlying pathogenic mechanisms for the individuals with variants with unknown effect, such as p.Arg698*. To assess this, we divided our cohort in two subgroups and (1) performed a structured genotype-phenotype analysis and (2) created and compared computationally modeled faces. Subgroup 1 ($n = 25$) included all probands carrying a predicted LOF variant (nonsense, frameshift or canonical splice-site, or balanced translocation) similar to variants p.Ser330* and c.1720+1G>T. Subgroup 2 ($n = 13$) comprised individuals with either missense variants or variants introducing a premature stop codon in the last exon of *TLK2*, such as p.Arg698*. Affected parents of probands with inherited mutations were not included in the subgroups. Next, we compared frequencies of 40 different features and frequencies of 15 facial dysmorphisms between the two groups via a two-tailed Fisher's exact test. This showed that both clinical features and facial dysmorphisms were remarkably similar between the two subgroups. From the 55 different features, none differed significantly between the two subgroups ($p < 0.05$), even without correction for multiple testing (Table S2). Second, averaged visualization of facial dysmorphism by computational modeling of 33 photographs from 22 individuals in subgroup 1 compared with 11 photographs from 8 individuals in subgroup 2 at different ages (Supplemental Subjects and Methods) showed consistent differences from a comparable number of gender- and age-matched controls, including blepharophimosis, telecanthus, broad nasal tip, and tall, pointed chin (Figures 3C and 3D). Given this strong overlap in phenotypes and facial dysmorphic features between probands with different type of mutations, it is likely that not only LOF variants but also the majority of identified missense variants and variants with a premature stop codon in the last exon have only a single functional copy of *TLK2*. Hence, we conclude that the predominant pathogenic mechanism of these *TLK2* mutations is haploinsufficiency.

Often mentioned together with TLK2 is its close interactor TLK1. From birth, murine *Tlk2* shows a similar expression pattern to the closely related paralog *Tlk1* across many tissues.[15] Human TLK1 has 84% identity to TLK2 at the protein level,[3] and it was shown that TLK1 depletion leads to extensive chromosome segregation defects in human cells.[17] Interestingly, *TLK1* (MIM: 608438) is (similarly to *TLK2*) intolerant for both missense and truncating mutations in healthy individuals (significant z-scores of 3.84 [*TLK1*] and 5.67 [*TLK2*] and pLI [constraint] scores of

1.00 for both TLK1 and TLK2) (ExAC database).[14] In the literature, four *de novo* variants have been reported in *TLK1* (GenBank: NM_012290.4): c.74C>T (p.Pro25Leu) in an individual with intellectual disability,[1] c.1697T>C (p.Met566Thr) in an individual with autism,[18] c.1796C>G (p.Ala599Gly) in an individual with a NDD and congenital heart disease,[19] and c.1101del (p.Lys367Asnfs*25) in an individual with schizophrenia.[20] Importantly, none of these variants are present in the ExAC or gnomAD databases. Taking this into account, it is possible that *TLK1* variants could contribute to NDDs, similar to the homolog *TLK2*. In future research, the exact role of *TLK1* in NDDs should be further explored.

In conclusion, we show that both *de novo* and inherited mutations in *TLK2* cause a distinct neurodevelopmental disorder, hallmarked by mild developmental delay, a variety of behavioral disorders, severe gastro-intestinal problems, and facial dysmorphism. The identification of a large number of individuals (n = 40, including two affected mothers) emphasizes the power and importance of data sharing, allowing us to delineate the clinical phenotype and to evaluate genotype-phenotype correlations. More than two-thirds of the individuals were identified in two relatively small countries: the Netherlands and the UK (Figure S1). With an estimated prevalence of ~1/566 (17/9,625) of *TLK2* variants in probands recruited to the DDD study, it is expected that a larger number of individuals with *TLK2* variants is present world-wide. In future, even more extensive data sharing than performed in this study will be needed to further extend the *TLK2* cohort. By analyzing three cell lines of affected individuals, we were able to confirm that at least two variants act through a heterozygous loss-of-function mechanism (haploinsufficiency). The phenotypes of these individuals and others with comparable loss-of-function variants significantly overlapped with phenotypes of individuals with other variant types, providing further evidence for the underlying disease mechanism of the *TLK2* variants. Given the genetic and functional similarities between TLK2 and TLK1, further research should focus on the potential role of *TLK1* mutations in developmental disorders.

Supplemental Data

Supplemental Data include one figure, seven tables, and Supplemental Subjects and Methods and can be found with this article online at https://doi.org/10.1016/j.ajhg.2018.04.014.

Acknowledgments

We thank H. Mlcochova, V.P. Sharma, and M. van Zeijl for technical support. We thank Sandra Yang for her help in contacting referring clinicians from GeneDx. This project was supported by the French Ministry of Health (DGOS) and the French National Agency for Research (ANR) (PRTS 2013 grant to C.S.-B.), the MRC through a Skills Development Fellowship (MR/R024952/1 to R.L.T.), Methodology Research Fellowship (MR/M014568/1 to C.N.), the Weatherall Institute of Molecular Medicine Strategic Alliance (G0902418, MC_UU_12025), the National Institute for Health Research (NIHR) Oxford Biomedical Research Centre Programme (A.O.M.W.), and Wellcome Investigator Award 102731 (A.O.M.W.). All research at Great Ormond Street Hospital NHS Foundation Trust and UCL Great Ormond Street Institute of Child Health is made possible by the NIHR Great Ormond Street Hospital Biomedical Research Centre. The DDD study presents independent research commissioned by the Health Innovation Challenge Fund (grant number HICF-1009-003), a parallel funding partnership between the Wellcome Trust and the Department of Health, and the Wellcome Trust Sanger Institute (grant number WT098051). The views expressed in this publication are those of the author(s) and not necessarily those of the Wellcome Trust or the Department of Health. The research team acknowledges the support of the NIHR, through the Comprehensive Clinical Research Network. This study makes use of DECIPHER, which is funded by the Wellcome Trust. ErasmusMC acknowledges Complete Genomics which donated 100 WGS trios for the centennial anniversary of the Erasmus University. J.A.C.G. was supported by the Innovation Fund (project number 2922). We acknowledge the HUGODIMS consortium, which was supported by a grant from the French Ministry of Health and from the Health Regional Agency from Poitou-Charentes (HUGODIMS, 2013, RC14_0107); we are grateful to Frédérique Allaire from the Health Regional Agency of Poitou-Charentes for supporting this project.

Declaration of Interests

The authors declare no competing interests.

Received: February 14, 2018
Accepted: April 26, 2018
Published: May 31, 2018

Web Resources

DECIPHER, https://decipher.sanger.ac.uk/
ExAC Browser, v.0.3.1, http://exac.broadinstitute.org/
GenBank, https://www.ncbi.nlm.nih.gov/genbank/
GeneMatcher, https://genematcher.org/
gnomAD Browser, v.r2.0.2, http://gnomad.broadinstitute.org/
OMIM, http://www.omim.org/

References

1. Lelieveld, S.H., Reijnders, M.R., Pfundt, R., Yntema, H.G., Kamsteeg, E.J., de Vries, P., de Vries, B.B., Willemsen, M.H., Kleefstra, T., Löhner, K., et al. (2016). Meta-analysis of 2,104 trios provides support for 10 new genes for intellectual disability. Nat. Neurosci. *19*, 1194–1196.
2. American Psychiatric Association (2013). Diagnostic and Statistical Manual of Mental Disorders, Fifth Edition (Washington, DC).
3. Silljé, H.H., Takahashi, K., Tanaka, K., Van Houwe, G., and Nigg, E.A. (1999). Mammalian homologues of the plant Tousled gene code for cell-cycle-regulated kinases with maximal activities linked to ongoing DNA replication. EMBO J. *18*, 5691–5702.
4. Yamakawa, A., Kameoka, Y., Hashimoto, K., Yoshitake, Y., Nishikawa, K., Tanihara, K., and Date, T. (1997). cDNA cloning and chromosomal mapping of genes encoding novel protein

kinases termed PKU-alpha and PKU-beta, which have nuclear localization signal. Gene *202*, 193–201.

5. Groth, A., Lukas, J., Nigg, E.A., Silljé, H.H., Wernstedt, C., Bartek, J., and Hansen, K. (2003). Human Tousled like kinases are targeted by an ATM- and Chk1-dependent DNA damage checkpoint. EMBO J. *22*, 1676–1687.
6. Silljé, H.H., and Nigg, E.A. (2001). Identification of human Asf1 chromatin assembly factors as substrates of Tousled-like kinases. Curr. Biol. *11*, 1068–1073.
7. Carrera, P., Moshkin, Y.M., Gronke, S., Sillje, H.H., Nigg, E.A., Jackle, H., and Karch, F. (2003). Tousled-like kinase functions with the chromatin assembly pathway regulating nuclear divisions. Genes Dev. *17*, 2578–2590.
8. Li, Y., DeFatta, R., Anthony, C., Sunavala, G., and De Benedetti, A. (2001). A translationally regulated Tousled kinase phosphorylates histone H3 and confers radioresistance when overexpressed. Oncogene *20*, 726–738.
9. Klimovskaia, I.M., Young, C., Strømme, C.B., Menard, P., Jasencakova, Z., Mejlvang, J., Ask, K., Ploug, M., Nielsen, M.L., Jensen, O.N., and Groth, A. (2014). Tousled-like kinases phosphorylate Asf1 to promote histone supply during DNA replication. Nat. Commun. *5*, 3394.
10. Bruinsma, W., van den Berg, J., Aprelia, M., and Medema, R.H. (2016). Tousled-like kinase 2 regulates recovery from a DNA damage-induced G2 arrest. EMBO Rep. *17*, 659–670.
11. Deciphering Developmental Disorders Study (2017). Prevalence and architecture of *de novo* mutations in developmental disorders. Nature *542*, 433–438.
12. Sobreira, N., Schiettecatte, F., Valle, D., and Hamosh, A. (2015). GeneMatcher: a matching tool for connecting investigators with an interest in the same gene. Hum. Mutat. *36*, 928–930.
13. Rahbari, R., Wuster, A., Lindsay, S.J., Hardwick, R.J., Alexandrov, L.B., Turki, S.A., Dominiczak, A., Morris, A., Porteous, D., Smith, B., et al.; UK10K Consortium (2016). Timing, rates and spectra of human germline mutation. Nat. Genet. *48*, 126–133.
14. Lek, M., Karczewski, K.J., Minikel, E.V., Samocha, K.E., Banks, E., Fennell, T., O'Donnell-Luria, A.H., Ware, J.S., Hill, A.J., Cummings, B.B., et al.; Exome Aggregation Consortium (2016). Analysis of protein-coding genetic variation in 60,706 humans. Nature *536*, 285–291.
15. Segura-Bayona, S., Knobel, P.A., González-Burón, H., Youssef, S.A., Peña-Blanco, A., Coyaud, É., López-Rovira, T., Rein, K., Palenzuela, L., Colombelli, J., et al. (2017). Differential requirements for Tousled-like kinases 1 and 2 in mammalian development. Cell Death Differ. *24*, 1872–1885.
16. Ishigaki, Y., Li, X., Serin, G., and Maquat, L.E. (2001). Evidence for a pioneer round of mRNA translation: mRNAs subject to nonsense-mediated decay in mammalian cells are bound by CBP80 and CBP20. Cell *106*, 607–617.
17. Hashimoto, M., Matsui, T., Iwabuchi, K., and Date, T. (2008). PKU-beta/TLK1 regulates myosin II activities, and is required for accurate equaled chromosome segregation. Mutat. Res. *657*, 63–67.
18. De Rubeis, S., He, X., Goldberg, A.P., Poultney, C.S., Samocha, K., Cicek, A.E., Kou, Y., Liu, L., Fromer, M., Walker, S., et al.; DDD Study; Homozygosity Mapping Collaborative for Autism; and UK10K Consortium (2014). Synaptic, transcriptional and chromatin genes disrupted in autism. Nature *515*, 209–215.
19. Homsy, J., Zaidi, S., Shen, Y., Ware, J.S., Samocha, K.E., Karczewski, K.J., DePalma, S.R., McKean, D., Wakimoto, H., Gorham, J., et al. (2015). De novo mutations in congenital heart disease with neurodevelopmental and other congenital anomalies. Science *350*, 1262–1266.
20. Fromer, M., Pocklington, A.J., Kavanagh, D.H., Williams, H.J., Dwyer, S., Gormley, P., Georgieva, L., Rees, E., Palta, P., Ruderfer, D.M., et al. (2014). De novo mutations in schizophrenia implicate synaptic networks. Nature *506*, 179–184.

The American Journal of Human Genetics, Volume *102*

Supplemental Data

De Novo and Inherited Loss-of-Function Variants in *TLK2*: Clinical and Genotype-Phenotype Evaluation of a Distinct Neurodevelopmental Disorder

Margot R.F. Reijnders, Kerry A. Miller, Mohsan Alvi, Jacqueline A.C. Goos, Melissa M. Lees, Anna de Burca, Alex Henderson, Alison Kraus, Barbara Mikat, Bert B.A. de Vries, Bertrand Isidor, Bronwyn Kerr, Carlo Marcelis, Caroline Schluth-Bolard, Charu Deshpande, Claudia A.L. Ruivenkamp, Dagmar Wieczorek, The Deciphering Developmental Disorders Study, Diana Baralle, Edward M. Blair, Hartmut Engels, Hermann-Josef Lüdecke, Jacqueline Eason, Gijs W.E. Santen, Jill Clayton-Smith, Kate Chandler, Katrina Tatton-Brown, Katelyn Payne, Katherine Helbig, Kelly Radtke, Kimberly M. Nugent, Kirsten Cremer, Tim M. Strom, Lynne M. Bird, Margje Sinnema, Maria Bitner-Glindzicz, Marieke F. van Dooren, Marielle Alders, Marije Koopmans, Lauren Brick, Mariya Kozenko, Megan L. Harline, Merel Klaassens, Michelle Steinraths, Nicola S. Cooper, Patrick Edery, Patrick Yap, Paulien A. Terhal, Peter J. van der Spek, Phillis Lakeman, Rachel L. Taylor, Rebecca O. Littlejohn, Rolph Pfundt, Saadet Mercimek-Andrews, Alexander P.A. Stegmann, Sarina G. Kant, Scott McLean, Shelagh Joss, Sigrid M.A. Swagemakers, Sofia Douzgou, Steven A. Wall, Sébastien Küry, Eduardo Calpena, Nils Koelling, Simon J. McGowan, Stephen R.F. Twigg, Irene M.J. Mathijssen, Christoffer Nellaker, Han G. Brunner, and Andrew O.M. Wilkie

Figure S1: Overview of countries where *TLK2* mutations in probands have been identified.

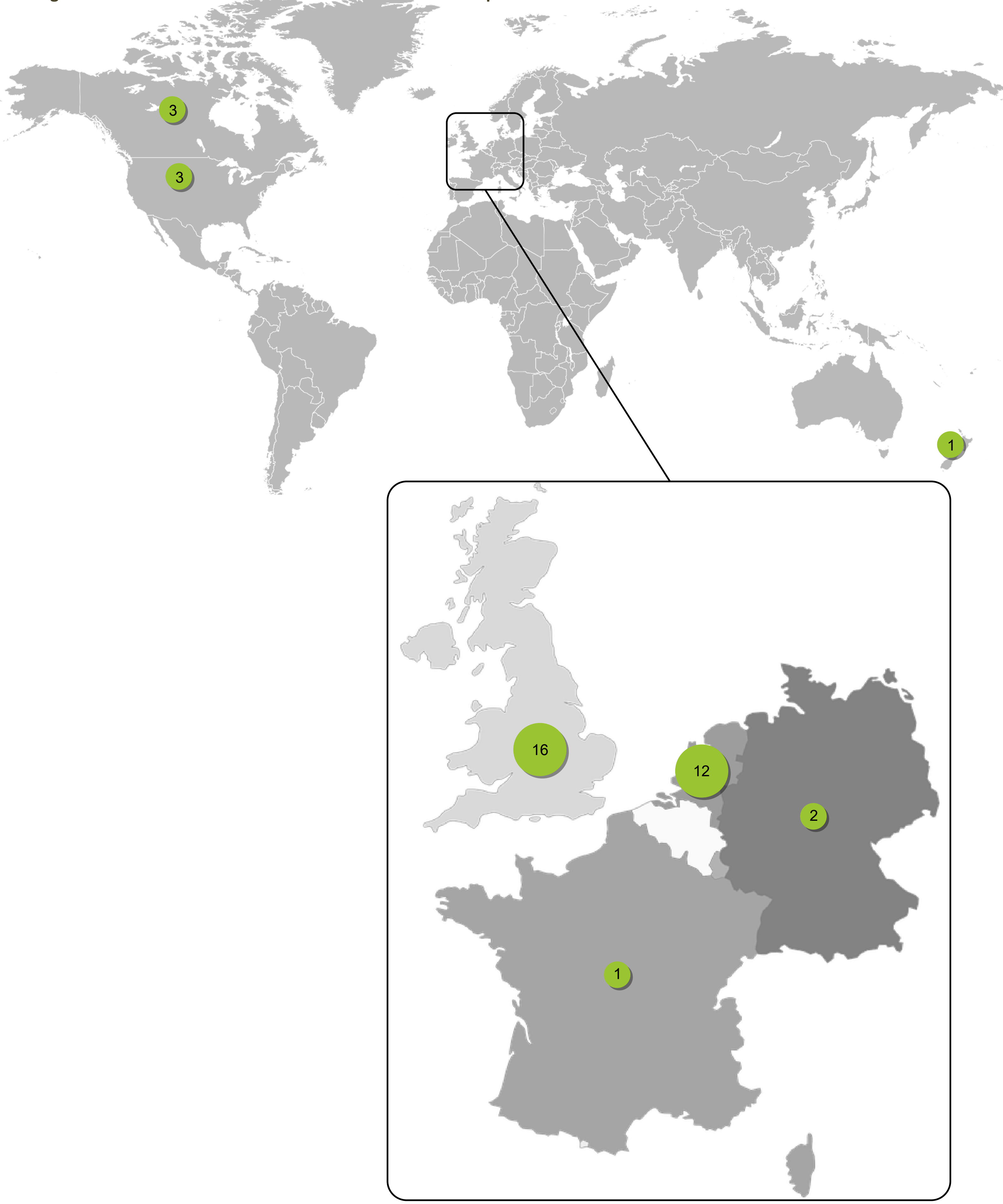

Table S1. Identified missense mutations in TLK2 (NM_006852) and predicted pathogenicity, domains, ExAC and gnomAD allele frequencies.

cDNA change	Amino acid change	PhyloP	SIFT	Mutation Taster	Polyphen	Domain	ExAC allele frequency	gnomAD allele frequency
c.890G>A	p.(Gly297Asp)	5.61	0.05 - Deleterious	Disease causing	1.000 - Probably damaging	None	0	0
c.1015C>T[1]	p.(Arg339Trp) [1]	6.18	0.04 - Deleterious	Disease causing	0.210 - Benign	Coiled coil motif	0	0
c.1016G>A [1]	p.(Arg339Gln) [1]	6.18	0.04 - Deleterious	Disease causing	0.998 - Probably damaging	Coiled coil motif	0	0
c.1273G>A	p.(Glu425Lys)	5.61	0.27 -Tolerated	Disease causing	1.000 - Probably damaging	Coiled coil motif	0	0
c.1412A>G	p.(His471Arg)	4.81	0.17 - Tolerated	Disease causing	1.000 - Probably damaging	Catalytic domain	0	0
c.1487A>G [2]	p.(His496Arg)[2]	4.73	0.12 - Tolerated	Disease causing	0.995 - Probably damaging	Catalytic domain	0	0
c.1636C>T	p.(Arg546Trp)	4.24	0 – Deleterious	Disease causing	0.683 – Possibly damaging	Catalytic domain	0	0.000004
c.1819G>A	p.(Asp607Asn)	6.02	0.65 – Tolerated	Disease causing	0.008 – Benign	Catalytic domain	0	0
c.1973C>G	p.(Pro658Arg)	6.02	0.65 – Tolerated	Disease causing	1.000 - Probably damaging	Catalytic domain	0	0

[1] Rows marked green indicate mutations affecting the same amino acid residue.
[2] Rows marked grey indicate recurrent mutations that were identified in two unrelated patients

Table S2. Frequencies of observed features and comparison between loss-of-function and other variant types.

Feature	Loss-of-function mutations (%)*	Missense variants (%)*	Fisher's Exact test	Total frequency (%)
Short stature	36	38	p=1.000	37
Low weight	13	15	p=1.000	14
Overweight	9	8	p=1.000	8
Microcephaly	24	23	p=1.000	24
Intellectual disability (ID)	70	83	p=0.450	74
Borderline ID	23	0	p=0.143	15
Low-normal development	5	9	p=1.000	6
Language delay	92	92	p=1.000	92
Motor delay	88	92	p=1.000	89
Behavior disorder	77	75	p=1.000	76
Autism spectrum disorder	23	50	p=0.138	32
Attention deficit - hyperactivity disorder (ADD/ADHD)	18	8	p=0.635	15
Anxiety	9	17	p=0.602	12
Tantrums	27	42	p=0.459	32
Aggressiveness	9	0	p=0.529	6
Obsessive compulsive disorder (OCD)	9	0	p=0.529	6
Social-emotional problems	18	17	p=1.000	18
Pica	4	0	p=1.000	3
Short attention span	4	8	p=1.000	5
Use of psychiatric drugs	25	23	p=1.000	24
Hypotonia	41	38	p=1.000	40
Epilepsy	17	8	p=0.638	14
Brain abnormality	27	20	p=1.000	25
Neonatal feeding difficulties	43	46	p=1.000	44
Constipation	55	69	p=0.488	60
Diarrhea	9	8	p=1.000	9
Refraction abnormality	33	27	p=1.000	31
Strabismus	22	42	p=0.258	29
Other eye abnormality	17	8	p=0.646	14
Craniosynostosis	13	8	p=1.000	11

Joint hypermobility	18	36	p=0.391	24
Scoliosis	9	8	p=1.000	9
Contractures hands	9	8	p=1.000	9
Walking on tiptoes	18	23	p=1.000	20
Pes planus	24	27	p=1.000	25
Hoarse voice	4	17	p=0.266	9
Plagiocephaly	21	8	p=0.394	16
Recurrent otitis media	30	20	p=0.686	27
Hypertrichosis	23	8	p=0.377	17
Conductive hearing loss	13	22	p=0.602	16
Sensorineural hearing loss	8	0	p=0.538	5
**Facial dysmorphisms**				
Asymmetric face	20	8	p=0.643	16
Microtia	36	15	p=0.268	29
Posteriorly rotated ears	32	23	P=0.714	29
Long face	29	25	p=1.000	27
Epicanthal folds	31	60	p=0.228	42
Blepharophimosis	80	85	p=1.000	82
Upward slanted palpebral fissures	44	77	p=0.086	55
Ptosis	24	15	p=0.689	21
Wide spaced eyes	80	62	p=0.263	74
Prominent nasal bridge	64	77	p=0.486	68
Broad nasal tip	60	50	p=0.473	66
High palate	21	50	p=0.303	30
Thin vermilion upper lip	68	50	p=0.470	62
Narrow mouth	24	50	p=0.146	32
Pointed, tall chin	44	38	p=1.000	42

* Frequencies in this table slightly differ from frequencies in the main text, since missing data (unknown presence of a feature) were excluded for statistical analysis. Clinical features of the two affected parents were not included in this analysis.

Table S3. Reported intracranial brain abnormalities.

	Description in MRI reports
1	Dilated 3rd and 4th lateral ventricles
2	Tonsillar herniation; deep sella; severe scalloping; narrow peripheral CSF spaces; prominent CSF surrounding optic nerve
3	Slightly small anterior pituitary
4	Mild-moderate volume loss of cerebral white matter
5	Simple gyration pattern; lack of white matter

Table S4. Overview of features reported in one or two patients.

Feature	Number of patients
Delayed bone age	1
Anterior fontanel closed before age of 3 months	1
Brachycephaly, not related to craniosynostosis	1
Single testicle, hypoplastic scrotum, hypospadias	1
Hypoplastic nails	1
Immune deficiency (low IgG and IgM)	1
Cryptorchidism	1
Laryngomalacia	1
Restless legs	1
Persistent ductus arteriosus	1
Precocious puberty	1
Rheumatoid arthritis	1
Pigmentary variant of iris	1
Right supernumerary nipple	1
Bilateral uveo-retinal colobomata	1
Left cranial nerve IV palsy	1
Fibromyalgia	1
Right inguinal hernia	1
Sensorineural hearing loss with asymmetrical widened vestibular aqueducts or semicircular canals	2

Table S5. Affected sutures in patients with craniosynostosis.

	**Suture**
1	Bicoronal
2	Right coronal and sagittal
3	Metopic
4	Coronal and metopic

Table S6. Primers used for the selective amplification of exons and flanking intron sequences from *TLK2.*

ID	Target length (bp)*	Forward primer	Reverse primer	Multiplex PCR Mix
TLK2-Ex2	345	ACACTGACGACATGGTTCTACAAATTACTGTGAGTTTTGTTCTACAG	TACGGTAGCAGAGACTTGGTCTACTATGTTAAATGACTACTGGAATGACC	4
TLK2-Ex3	284	ACACTGACGACATGGTTCTACAACGCCATTGTATTCCAGCCGGGGTGAT	TACGGTAGCAGAGACTTGGTCTCAGCCTTGAGCCACCAAACCTGGCCCAAAC	3
TLK2-Ex4	401	ACACTGACGACATGGTTCTACATAAGAGGAAGACAGTGATTCAGGAC	TACGGTAGCAGAGACTTGGTCTGAACTAACACTGTTCTGTCAGGTG	1
TLK2-Ex5	435	ACACTGACGACATGGTTCTACATGGAGGAAATAGTCTGTTCTTG	TACGGTAGCAGAGACTTGGTCTATGTTGCCCAGGTTGGCCTCGAACT	5
TLK2-Ex6	337	ACACTGACGACATGGTTCTACAGCATAGTACTGTTTTGAATTATTCATATCG	TACGGTAGCAGAGACTTGGTCTCTCTTCTGTAAAAAGCTAATTTACTGAC	2
TLK2-Ex7	314	ACACTGACGACATGGTTCTACACTTATATTTGATAACTGTTTTTAACCCG	TACGGTAGCAGAGACTTGGTCTGAGCACTAGGGCAATGGAAAGGATA	2
TLK2-Ex8	415	ACACTGACGACATGGTTCTACAGAACTTGGTATAAACCACCATGTCC	TACGGTAGCAGAGACTTGGTCTGTGGTCAGAGAAATACAGAGAAGTC	2
TLK2-Ex9	245	ACACTGACGACATGGTTCTACAATTTGTGTGAGCAAGTGCTTTTTCC	TACGGTAGCAGAGACTTGGTCTGGTGCTTGCTATAAAATCTCTTACA	4
TLK2-Ex10	473	ACACTGACGACATGGTTCTACAAAACATGCCCAAATTAGTAATTCAA	TACGGTAGCAGAGACTTGGTCTCAAATCATGTTCCTAAAAAGCTCTAC	6
TLK2-Ex11	368	ACACTGACGACATGGTTCTACATTCTAAGAAGTGTCTTTATCCATGC	TACGGTAGCAGAGACTTGGTCTAGGACTTCACCTCATTCGATAC	3
TLK2-Ex12	325	ACACTGACGACATGGTTCTACAAAATTGGATACACAAGTGACAAATTG	TACGGTAGCAGAGACTTGGTCTCTATTGCCGGTGACAATCAAC	5
TLK2-Ex13	392	ACACTGACGACATGGTTCTACAGCTTTGAAGTTCTTCCCTCACATC	TACGGTAGCAGAGACTTGGTCTCACTGAAGCTTTCTGCTGCTATG	5
TLK2-Ex14	338	ACACTGACGACATGGTTCTACATTACTGAACTCCTCTGTATGGTTTG	TACGGTAGCAGAGACTTGGTCTAGCAATCTCCAACCCAATATGC	4
TLK2-Ex15	451	ACACTGACGACATGGTTCTACACTGGGAATTTTGCAAGCGTGG	TACGGTAGCAGAGACTTGGTCTTATGAGGCAGGAAGTACAGAACC	2
TLK2-Ex16	350	ACACTGACGACATGGTTCTACATAATCACAAGTTTCAAGAAGGTGCT	TACGGTAGCAGAGACTTGGTCTACCAACAACAATGCACGTAAAG	6
TLK2-Ex17	409	ACACTGACGACATGGTTCTACATCTCAATGGCTTGGTAGATTCC	TACGGTAGCAGAGACTTGGTCTTGTCACAAATTACTTGGTTCCCTC	1
TLK2-Ex18	421	ACACTGACGACATGGTTCTACAAGGTAGTGTTAATCTGCTTGCTC	TACGGTAGCAGAGACTTGGTCTTCCAACACGCCCTCCTAAAC	3
TLK2-Ex19	329	ACACTGACGACATGGTTCTACAAGTCCAGATTGCTTGATTCCC	TACGGTAGCAGAGACTTGGTCTGCCACATCTCTATAGCCAACCTG	6
TLK2-Ex20	368	ACACTGACGACATGGTTCTACAGTACATGTCTTAAACTTATATGATC	TACGGTAGCAGAGACTTGGTCTCCTAGGGTTGAGGATTTCTGCT	-
TLK2-Ex21	431	ACACTGACGACATGGTTCTACACCCACTCTGCTTTGACCTGGTAG	TACGGTAGCAGAGACTTGGTCTTTCACACTGAAGAATCCATCCA	6
TLK2-Ex22	452	ACACTGACGACATGGTTCTACAAGAGGTACTTCTGTTGGTGCTT	TACGGTAGCAGAGACTTGGTCTGGATTCGCTATGTTCCAAACC	1

*excluding universal adaptors CS1 and CS2 (underlined in the primer sequences; 22 nt each).

Table S7. Primers used for analysis of *TLK2* cDNA.

TLK2 mutation	Forward primer (5'→3')	Reverse primer (5'→3')	Target length (bp)*	Restriction enzyme
c.989C>A p.(S330*)	ACACTGACGACATGGTTCTACATGCAAGACCGCTTGAGACTG	TACGGTAGCAGAGACTTGGTCTCAGCTCTGCCTGGATCTCTG	347	ApoI
c.1720+1G>T	ACACTGACGACATGGTTCTACAGCATGCATGTAGGGAATACCG	TACGGTAGCAGAGACTTGGTCTATCTCTCCACACGCTGTACC	300	-
c.2092C>T p.(R698*)	ACACTGACGACATGGTTCTACAGCATGCATGTAGGGAATACCG	TACGGTAGCAGAGACTTGGTCTACTGTTATTGGACGCCCCAG	781	Hpy99I

*excluding universal adaptors CS1 and CS2 (underlined in the primer sequences; 22 nt each)

SUPPLEMENTAL METHODS

1. Identification of TLK2 variants

Overview

**Method**	**Setting**	**Number of individuals**
WES	Diagnostics	18 probands
WES (A)	Research	18 probands + 2 affected parents
WGS (B)	Research	2 probands

A) Whole exome sequencing in research settings (*n*=18+2)

Oxford study (*n*=1)

WES was performed as part of a study of seven unrelated individuals with bicoronal synostosis as described by Sharma *et al.*[1] Initial filtering, performed as described[1] did not highlight any strong candidate variant, but after comparison with WGS data from another craniosynostosis cohort (individual 2, WGS in research settings, see below), the *TLK2* c.989C>A, p.(S330*) variant was identified and prioritized for further analysis. The study has ethical approval from Oxfordshire Research Ethics Committee B (reference C02.143), and London Riverside Research Ethics Committee (reference 09/H0706/20).

DDD study (*n*=15 probands +2 affected parents)

Following identification of *TLK2* mutations in the Rotterdam and Oxford studies, rare *TLK2* variants were requested from the Deciphering Developmental Disorders (DDD) research study (http://www.ddduk.org)[2; 3] as part of the approved Complementary Analysis Project #144. Data were initially obtained from Datafreeze 2 and later extended to Datafreeze 3, comprising 7,833 trios and 1,792 singletons with undiagnosed developmental disorders, primarily developmental delay/ learning disability. Non-synonymous variants absent from ExAC in 17 unrelated probands were prioritized and the referring clinician contacted, requesting further clinical information and DNA for confirmatory dideoxy-sequencing. Two cases had a mutation inherited from an affected parent. Two further cases were excluded from the analysis, one because a co-existing nonsense mutation in *KIF11* confounded the phenotypic interpretation and the other because the parents could not be re-contacted. The study has UK Research Ethics Committee approval (10/H0305/83, granted by the Cambridge South REC, and GEN/284/12 granted by the Republic of Ireland REC).

MRBE study (*n*=2)

WES and filtering for rare *de novo* variants was performed as published previously by Schäfgen et al.[4] as part of a study on 311 individuals with ID / developmental delay (DD) with or without additional features (e.g. craniofacial dysmorphism, organ malformation etc.) that could not be attributed to a clinically recognisable syndrome by experienced clinical geneticists. All investigations were performed in accordance with the Declaration of Helsinki and were approved by the local institutional review boards (Ethics Committee of the Medical Faculty of the University of Bonn, approvals 131/08 and 024/12, Ethics Committee of the Medical Faculty of the University of Essen 08-3663).

<u>HUGODIMS study</u>

WES was performed as part of the research program conducted by the Western France consortium HUGODIMS (French acronym standing for "Projet inter-régional Français des Hôpitaux Universitaires du Grand Ouest pour l'exploration par approche exomique des causes moléculaires de Déficience Intellectuelle isolée ou syndromique Modérée à Sévère").[5] Two *de novo* candidate missense variants were highlighted, in *GABRA1* and *TLK2.* Although the *TLK2* variant is likely to contribute to the phenotype of this patient, a contribution of the *GABRA1* variant cannot be excluded. Therefore, this patient was excluded from further phenotypic analysis. All families gave written informed consent for inclusion in the study which has been approved by the CHU de Nantes-ethics committee (number CCTIRS: 14.556).

B) Whole genome sequencing in research settings (*n*=2)

<u>Individual 1: Breakpoint mapping of balanced translocation by WGS</u>

Standard RHG and GTG karyotype identified an apparently balanced reciprocal translocation 46,XX,t(4;17)(q27;q23). Array-CGH did not identify any pathogenic imbalances (Human Genome CGH microarray 180K, Agilent, Santa Clara, CA, USA). A whole genome library was prepared with 3 µg genomic blood DNA following the Illumina TruSeq protocol (Illumina, San Diego, California, USA). A 350 bp fragment library was sequenced on an Illumina NextSeq 500 as paired-end 101 bp reads. The sequencing depth was 8.71x. Alignment of the reads against the GRCh37 version of the human genome was done using BWA-MEM v 0.7.10.[6] The reads were then sorted using Samtools v 1.3.1[7], and the duplicates removed by PicardTools v 1.138 (picard.sourceforge.net). Then, the structural variants (SV) were detected using BreakDancer v 1.4.5[8] and annotated using an in-house script, mainly for the purpose of filtering them on the basis of their occurrence in a local database. Integrative Genomics Viewer v 2.3 was used for the SV visualization.[9]

In order to amplify junction fragments, primer pairs were selected on each side of the breakpoint region delimited by WGS (primers sequence available on request). PCR amplification was performed using the Taq DNA Core kit 25 (MP Biomedicals, Solon, Ohio). DNA from a non-carrier of the chromosomal rearrangement was amplified as a negative control. Specific PCR products corresponding to the junction fragment were sequenced by the dideoxy method.

Individual 2: WGS

WGS was performed on DNA of the proband and his parents by Complete Genomics, a BGI company (Mountain View, CA, USA) as described by Drmanac et al..[10] Variants were annotated using GRCh37/hg19 and dbSNP build 130. Data were analyzed using cga tools version 1.6.0.43 and TIBCO Spotfire 7.0.0 (TIBCO Software Inc., Boston, MA, USA). A *de novo* disease model was tested using the calldiff script (Python script kindly provided by Complete Genomics) as described by Gilissen et al.,[11] which identified 76 *de novo* high score variants (both somatic scores ≥5), with only one that was protein affecting (c.907C>T in *TLK2*). Also, *de novo* X-linked variants were excluded. The study has approval of the Medical Ethical Committee of the Erasmus University Medical Center Rotterdam (MEC-2012-140 and MEC-2013-547).

2. Confirmation of research variants

All variants identified in *TLK2* were numbered according to Genbank accession NM_006852.3 (ENST00000346027.9), a transcript comprising 22 exons that encodes a 750 amino acid protein. An alternative transcript containing an additional 66 nucleotide exon between exons 12 and 13 has been annotated (NM_001284333), but the additional exon is poorly conserved in mammalian species and its physiological significance is uncertain. Confirmation of genetic variants identified in *TLK2* by research exome or whole-genome sequencing was carried out by dideoxy-sequencing of genomic PCR amplification products. Amplification of products utilized primers shown in Table S1; sequencing and visualization of variants was performed as previously described.[12]

3. Screening of individuals with craniosynostosis

We screened all 22 coding exons including exon/intron boundaries of *TLK2* in a mixed cohort of craniosynostosis patients using primers optimized for amplification of *TLK2* only (to ensure no amplification of unprocessed or processed partial pseudogenes located on chromosomes 4, 7, 10 and 17). Primers were designed to amplify target regions between 245-473 bp with the addition of CS1 (5’-ACACTGACGACATGGTTCTACA-3’) and CS2 (5’-TACGGTAGCAGAGACTTGGTCT-3’) adaptor sequences included on the 5’ ends of all target-specific forward and reverse primers, respectively. Briefly, primers were multiplexed into pools of 3-4 optimized pairs (with the exception of exon 20,

which was amplified independently), with a final concentration of 0.5 µM per primer in a reaction volume of 20 µl, containing 1x Buffer, 200 µM each dNTP and 0.5 U of Q5® High-Fidelity DNA Polymerase (New England BioLabs). Cycling conditions consisted of a 30 s denaturation step at 98°C, followed by 35 cycles of 98°C for 10 s, 60°C for 30 s and 72°C for 30 s, and a final extension step of 72°C for 10 min. Illumina-specific sequence adaptors and 10 bp sample indexes were attached using the Access Array™ Barcode Library for Illumina® Sequencers-384, Single Direction (Fluidigm) at a concentration of 0.4 µM per primer with Q5® High-Fidelity DNA Polymerase as above, for 9 cycles only. Primer/adaptor sequences and details of multiplex pools are provided in Table S1.

Indexed PCR products were pooled, purified with AxyPrep MAG PCR Clean-Up Kit (Axygen) and quantified using the 2200 TapeStation (Agilent Technologies) with a High Sensitivity D1000 ScreenTape and a Qubit® 1.0 Fluorometer (ThermoFisher Scientific), following the manufacturer's instructions. Pooled and indexed PCR products were diluted to a final concentration of 9 pM and sequenced using the Illumina MiSeq platform with a MiSeq Reagent Kit v2 for 500 cycles (Illumina) according to the manufacturer's instructions. All targets of *TLK2* were sequenced with a minimum coverage of 10 reads in a total of 309 samples from individuals with mixed types of craniosynostosis without a defined genetic cause.

4. Analysis of TLK2 Splicing

Skin fibroblasts from an individual heterozygous for the *TLK2* c.989C>A, p.(Ser330*) mutation were cultured in Dulbecco modified Eagle medium (DMEM) supplemented with 10% fetal bovine serum (FBS) and 1% penicillin and streptomycin. Lymphoblastoid cell lines were cultured in RPMI-1640 supplemented with 15% FBS and 1% penicillin and streptomycin. All reagents were purchased from Life Technologies unless otherwise stated. Patient cells were cultured in two concurrent T25 flasks, incubated with either 100 ng cycloheximide or 1% DMSO. Flasks were incubated at 37°C with 5% CO_2 for 4 h, then resuspended in Trizol for RNA extraction. Total RNA was extracted using the Direct-zol™ RNA MiniPrep Kit (Zymo Research) according to the manufacturer's instructions. gDNA was removed by treatment with DNase I (Sigma-Aldrich), following the manufacturer's protocol. cDNA was generated from 1 µg RNA with the addition of 0.1 µg random oligomers and 0.25 µg oligo dT primers using the RevertAid First Strand cDNA Synthesis kit (ThermoFisher Scientific). The reaction mixture was incubated at 65°C for 5 min, chilled on ice prior to addition of other reaction components, then incubated for 5 min at 25°C for 5 min followed by 60 min at 42°C before being terminated by heating to 70°C for 5 min. Primers used for RT-PCR and deep sequencing are shown in Table S2 (note, the CS1 and CS2 tags were omitted for agarose gel visualization but included for deep sequencing).

Transcripts were analyzed by gel electrophoresis of cDNA digested with a restriction enzyme targeting the wild-type transcript. The aberrant splice product obtained from the patient with c.1720+1G>T mutation was extracted using the High Pure PCR Product Purification Kit (Roche) and dideoxy-sequencing was performed as above. cDNA was amplified in triplicate for deep sequencing and indexed as above with a minimum coverage of 332 at the variant position.

5. Bioinformatic analysis of deep sequencing data

Variant calls, allele counts and coverage information were obtained with amplimap (https://github.com/koelling/amplimap), a pipeline built using *Snakemake* version 3.11.2.[13] Reads were trimmed to remove primers and aligned to GRCh37 (without alt contigs) using *bwa mem* version 0.7.12[14] for genomic DNA and *STAR* version 2.5.1b[15] for cDNA. For the allele balance analysis the two target SNPs were masked to prevent reference bias during mapping. Coverage was calculated using *bedtools* version 2.25.0[16], requiring a minimum coverage of 10 to consider a target region fully covered. Variants were called with *Platypus* version 0.8.1[17] and annotated using *Annovar* version 2015-06-17[18]. Further processing and annotation was performed in Python 3.5.3 with *pysam*[19], *biopython*[20] and *pandas.*[21]

6. Computational analysis of facial photographs

To visualise the characteristic facial features of groups of subjects, we generate realistic, de-identified average faces. We used a fully-automated algorithm that (1) annotates a face with a constellation of 68 facial feature points, (2) creates an average face mesh and (3) morphs the face of each subject onto the average face mesh. The face averaging algorithm was developed from previous work,[22; 23] and improved upon to produce more visually appealing, and better de-identified averages. We used an ensemble of regression trees to detect a constellation of 68 feature points on the face.[24] The face mesh of each patient was aligned to a target mesh, which was created from healthy control patients, using the feature points along the middle of the face. The average face was created by morphing the image of each patient’s face onto the average face mesh. To avoid biases towards individuals with multiple images, each patient's contribution to the average face mesh was equally weighted. Finally, to avoid variances in illumination between images, we normalized the pixel values within the face to an average value across all faces for each average.

SUPPLEMENTAL REFERENCES

1. Sharma, V.P., Fenwick, A.L., Brockop, M.S., McGowan, S.J., Goos, J.A., Hoogeboom, A.J., Brady, A.F., Jeelani, N.O., Lynch, S.A., Mulliken, J.B., et al. (2013). Mutations in TCF12, encoding a basic helix-loop-helix partner of TWIST1, are a frequent cause of coronal craniosynostosis. Nat Genet 45, 304-307.
2. Deciphering Developmental Disorders Study. (2015). Large-scale discovery of novel genetic causes of developmental disorders. Nature 519, 223-228.
3. Deciphering Developmental Disorders Study. (2017). Prevalence and architecture of de novo mutations in developmental disorders. Nature 542, 433-438.
4. Schafgen, J., Cremer, K., Becker, J., Wieland, T., Zink, A.M., Kim, S., Windheuser, I.C., Kreiss, M., Aretz, S., Strom, T.M., et al. (2016). De novo nonsense and frameshift variants of TCF20 in individuals with intellectual disability and postnatal overgrowth. Eur J Hum Genet 24, 1739-1745.
5. Isidor, B., Kury, S., Rosenfeld, J.A., Besnard, T., Schmitt, S., Joss, S., Davies, S.J., Lebel, R.R., Henderson, A., Schaaf, C.P., et al. (2016). De novo truncating mutations in the kinetochore-microtubules attachment gene CHAMP1 Cause Syndromic Intellectual Disability. Hum Mutat 37, 354-358.
6. Li, H. (2013) Aligning sequence reads, clone sequences and assembly contigs with BWA-MEM. arXiv preprint arXiv:13033997.
7. Li, H., Handsaker, B., Wysoker, A., Fennell, T., Ruan, J., Homer, N., Marth, G., Abecasis, G., Durbin, R., and Genome Project Data Processing, S. (2009). The Sequence Alignment/Map format and SAMtools. Bioinformatics 25, 2078-2079.
8. Chen, K., Wallis, J.W., McLellan, M.D., Larson, D.E., Kalicki, J.M., Pohl, C.S., McGrath, S.D., Wendl, M.C., Zhang, Q., Locke, D.P., et al. (2009). BreakDancer: an algorithm for high-resolution mapping of genomic structural variation. Nat Methods 6, 677-681.
9. Thorvaldsdottir, H., Robinson, J.T., and Mesirov, J.P. (2013). Integrative Genomics Viewer (IGV): high-performance genomics data visualization and exploration. Brief Bioinform 14, 178-192.
10. Drmanac, R., Sparks, A.B., Callow, M.J., Halpern, A.L., Burns, N.L., Kermani, B.G., Carnevali, P., Nazarenko, I., Nilsen, G.B., Yeung, G., et al. (2010). Human genome sequencing using unchained base reads on self-assembling DNA nanoarrays. Science 327, 78-81.
11. Gilissen, C., Hehir-Kwa, J.Y., Thung, D.T., van de Vorst, M., van Bon, B.W., Willemsen, M.H., Kwint, M., Janssen, I.M., Hoischen, A., Schenck, A., et al. (2014). Genome sequencing identifies major causes of severe intellectual disability. Nature 511, 344-347.
12. Miller, K.A., Twigg, S.R., McGowan, S.J., Phipps, J.M., Fenwick, A.L., Johnson, D., Wall, S.A., Noons, P., Rees, K.E., Tidey, E.A., et al. (2017). Diagnostic value of exome and whole genome sequencing in craniosynostosis. J Med Genet 54, 260-268.
13. Koster, J., and Rahmann, S. (2012). Snakemake--a scalable bioinformatics workflow engine. Bioinformatics 28, 2520-2522.
14. Li, H., and Durbin, R. (2009). Fast and accurate short read alignment with Burrows-Wheeler transform. Bioinformatics 25, 1754-1760.
15. Dobin, A., Davis, C.A., Schlesinger, F., Drenkow, J., Zaleski, C., Jha, S., Batut, P., Chaisson, M., and Gingeras, T.R. (2013). STAR: ultrafast universal RNA-seq aligner. Bioinformatics 29, 15-21.

16. Quinlan, A.R., and Hall, I.M. (2010). BEDTools: a flexible suite of utilities for comparing genomic features. Bioinformatics 26, 841-842.
17. Rimmer, A., Phan, H., Mathieson, I., Iqbal, Z., Twigg, S.R.F., Consortium, W.G.S., Wilkie, A.O.M., McVean, G., and Lunter, G. (2014). Integrating mapping-, assembly- and haplotype-based approaches for calling variants in clinical sequencing applications. Nat Genet 46, 912-918.
18. Wang, K., Li, M., and Hakonarson, H. (2010). ANNOVAR: functional annotation of genetic variants from high-throughput sequencing data. Nucleic Acids Res 38, e164.
19. Heger A, B.T., Finkernagel F, Goodstadt L, Goodson M, Jacobs KB, Lunter G, Martin M, Schiller B. Pysam: Python Interface for the SAM/BAM Sequence Alignment and Mapping Format. https://githubcom/pysam-developers/pysam Accessed 18-08-2017.
20. Cock, P.J., Antao, T., Chang, J.T., Chapman, B.A., Cox, C.J., Dalke, A., Friedberg, I., Hamelryck, T., Kauff, F., Wilczynski, B., et al. (2009). Biopython: freely available Python tools for computational molecular biology and bioinformatics. Bioinformatics 25, 1422-1423.
21. McKinney, W. (2010). Data structures for statistical computing in Python. in Proceedings of the 9th Python in Science Conference, 51-56.
22. Ferry, Q., Steinberg, J., Webber, C., FitzPatrick, D.R., Ponting, C.P., Zisserman, A., and Nellaker, C. (2014). Diagnostically relevant facial gestalt information from ordinary photos. eLife 3, e02020.
23. Ansari, M., Poke, G., Ferry, Q., Williamson, K., Aldridge, R., Meynert, A.M., Bengani, H., Chan, C.Y., Kayserili, H., Avci, S., et al. (2014). Genetic heterogeneity in Cornelia de Lange syndrome (CdLS) and CdLS-like phenotypes with observed and predicted levels of mosaicism. J Med Genet 51, 659-668.
24. Kazemi, V., Sullivan, J. (2014). One millisecond face alignment with an ensemble of regression trees. Proceedings of the IEEE Computer Society Conference on Computer Vision and Pattern Recognition, 1867–1874.
